# Variable Combinations of Specific Ephrin Ligand/Eph Receptor Pairs Control Embryonic Tissue Separation

**DOI:** 10.1371/journal.pbio.1001955

**Published:** 2014-09-23

**Authors:** Nazanin Rohani, Andrea Parmeggiani, Rudolf Winklbauer, François Fagotto

**Affiliations:** 1 Department of Biology, McGill University, Montreal, Quebec, Canada; 2 Complex Systems and Non Linear Phenomena, L2C–UMR 5221 CNRS–UM2, Montpellier, France; 3 Biological Physics and Systems Biology, DIMNP–UMR 5235 CNRS–UM2 et 1, Montpellier, France; 4 Department of Cell and Systems Biology, University of Toronto, Toronto, Canada; Osaka University, Japan

## Abstract

Ephrins and Eph receptors are involved in the establishment of vertebrate tissue boundaries. The complexity of the system is puzzling, however in many instances, tissues express multiple ephrins and Ephs on both sides of the boundary, a situation that should in principle cause repulsion between cells within each tissue. Although co-expression of ephrins and Eph receptors is widespread in embryonic tissues, neurons, and cancer cells, it is still unresolved how the respective signals are integrated into a coherent output. We present a simple explanation for the confinement of repulsion to the tissue interface: Using the dorsal ectoderm–mesoderm boundary of the Xenopus embryo as a model, we identify selective functional interactions between ephrin–Eph pairs that are expressed in partial complementary patterns. The combined repulsive signals add up to be strongest across the boundary, where they reach sufficient intensity to trigger cell detachments. The process can be largely explained using a simple model based exclusively on relative ephrin and Eph concentrations and binding affinities. We generalize these findings for the ventral ectoderm–mesoderm boundary and the notochord boundary, both of which appear to function on the same principles. These results provide a paradigm for how developmental systems may integrate multiple cues to generate discrete local outcomes.

## Abstract 2

Vertebrate embryonic cells recognize self from non-self, thus restricting repulsion at tissue boundaries, through a combination of multiple ephrins and Eph receptors, simply based on binding selectivity and asymmetric expression.

## Author Summary

How embryonic tissues separate from each other to shape the developing organism is a fundamental question in developmental biology. In vertebrates, this process relies on local repulsive reactions specifically generated at contacts between cells of different types. These reactions are triggered by typical repulsive cell surface cues, the ephrin ligands, and Eph receptors. However, the expression of multiple ephrins and the Eph receptors by each cell type represents a puzzle: Why is repulsion observed only at the tissue interface and not within the tissue itself? By studying three cases of separation in the early amphibian embryo, we uncover a surprisingly simple logic underlying this phenomenon, which can be explained by the selectivity of ligand–receptor interactions and by their asymmetric distribution. The system is set such that, despite generalized interactions throughout the tissues, it is only at contacts between different cell types that the overall repulsive output is sufficiently strong to overcome cell–cell adhesion. Our study may serve as paradigm for how systematic dissection of complex cellular systems can reduce them to simple laws and make them intelligible.

## Introduction

In vertebrates, ephrins and Eph receptors have emerged as major players in the formation of cleft-like tissue boundaries. They control segmentation of rhombomeres [1] and somites [2],[3] and the separation of embryonic germ layers [4]–[6]. Ephrins as well as Eph receptors are divided into A and B subclasses, based on their structural and binding characteristics. They are considered to bind promiscuously within each subclass, ephrinAs with EphAs and ephrinBs with EphBs [7], with the exceptions of EphA4, which can interact with both ephrinAs and Bs, and EphB2, which can bind ephrinA5 [8]–[10]. Classically, a single ephrin–Eph pair is expressed in a complementary pattern in adjacent tissues. However, in many physiological situations, each cell type may express multiple ephrins and Eph receptors [11],[12]. To explain the restriction of signaling to the tissue boundary, one must assume that these molecules interact in more selective ways. Consistently, in vitro studies have yielded a wide range of binding affinities between various ephrins and Eph receptors, suggesting a substantial degree of specificity, but the biological significance of these differences has not been clearly established [11],[13],[14]. Moreover, the presence of ephrins and Ephs in the same cell introduces a whole additional layer of complexity involving effects such as ephrin–Eph cis-interactions [15],[16] as well as potential cross-talks between the downstream signaling events [10],[17].

Understanding how the global output is determined under in vivo conditions has thus remained a daunting challenge. An example of where the integration of multiple co-expressed Eph receptors and ephrins can be tested is the ectoderm/mesoderm boundary in the early Xenopus embryo. We have demonstrated that ephrins and Ephs act directly at the tissue interface, where they generate cycles of attachments and detachments through transient activation of Rho GTPases [4]. This mechanism based on cell contact-mediated repulsion is highly reminiscent of neuronal contact guidance and utilizes the same molecular cues [18]. We showed that full separation required antiparallel forward signaling across the boundary such that ephrins in the mesoderm stimulate Ephs in the ectoderm and vice versa [4]. This observation was quite puzzling, as ephrin and Eph should in principle interact equally between cells within each tissue, which should cause repulsion and eventually lead to tissue dissociation. We ask here how cell repulsion is restricted to sites of contacts between the two tissues.

## Results

### Asymmetric Expression of Specific Ephrins and Ephs Is Required at the Dorsal Ectoderm–Mesoderm Boundary

**Figure 1:**
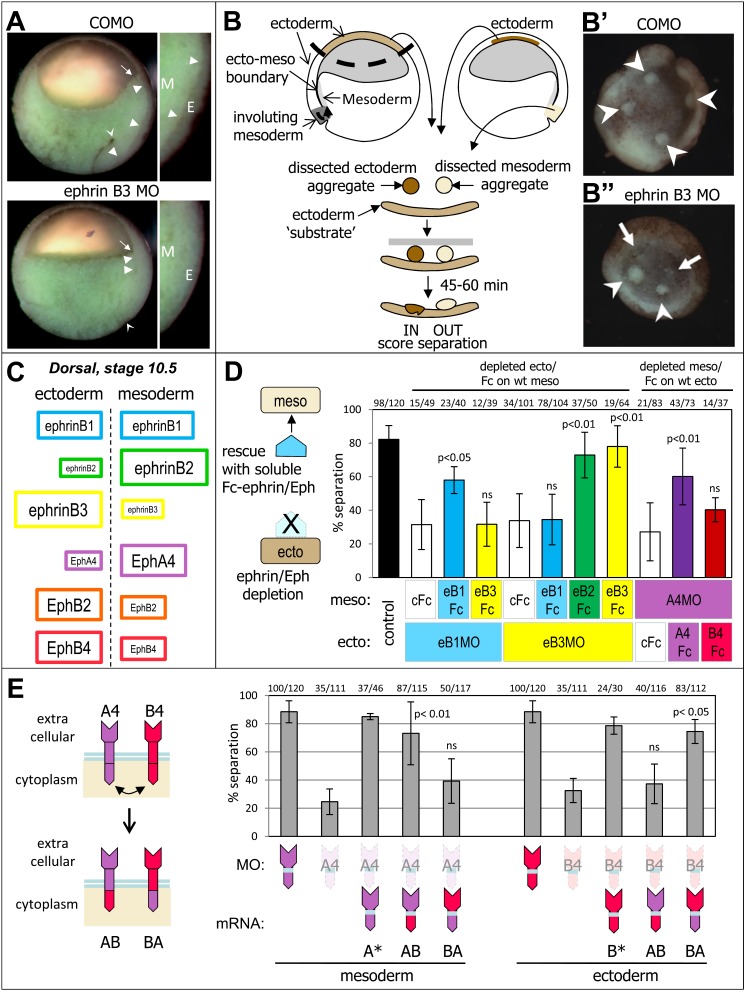
Multiple ephrins and Eph receptors are specifically required for ectoderm–mesoderm separation. (A) Ephrin–Eph signaling is required for ectoderm–mesoderm separation. Sagittal section of gastrula embryos, injected with antisense MOs. Inserts show detail of the dorsal side. The boundary separating ectoderm (E) from mesoderm (M) is marked by arrowheads. The concave arrowhead indicates the position of bottle cells, and the arrow the anterior edge of the anterior mesoderm. COMO, control morpholinos; EphrinB3 MO, The boundary is largely absent. Arrowheads point to a fuzzy remnant of ectoderm–mesoderm interface. Mesoderm involution is strongly impaired (arrow and concave arrowhead). (B) Diagram of the early Xenopus gastrula and of the in vitro tissue separation assay. In this assay, test explants obtained by dissection from the inner layer of the ectoderm or from the involuting mesoderm (blastopore lip) are placed on an ectoderm substrate. Ectoderm explants rapidly integrate into the substrate, whereas mesoderm aggregates remain well separated. The percentage of explants remaining separate is scored after 45–60 min. (B′) Ectoderm injected with ephrinB3 MO fails to maintain separation and incorporate mesoderm explants (arrows). Arrowheads, explants remaining separated. (C) Asymmetric expression of multiple ephrins and Ephs across the ectoderm–mesoderm boundary. Schematic representation of the relative expression of ephrins and Eph receptors analyzed in this study, based on RT-qPCR (Figure S1A). The same color code is used in all figures. (D) Each ephrin/Eph is specifically required. In vitro tissue separation assay. Depletion of ephrinB1 or ephrinB3 in the ectoderm (eB1MO, eB3MO) or EphA4 in the mesoderm (A4MO) led to inhibition of separation. Separation could be restored by treating directly the surface of the other tissue explant for 15 min with soluble Fc fragments of the corresponding ephrin/Eph molecule. Other ephrins/Ephs failed to rescue, with the exception of eB2, which could rescue eB3 depletion. cFc, control anti-human Fc antibody. Numbers on top indicate ratios of separated explants to total number of explants. *p* values = Student's *t* test. (E) The specific role of Eph receptors requires their extracellular domain, but the cytoplasmic tails are interchangeable. Chimeras were designed in which cytoplasmic domains of EphA4 and EphB4 were swapped. Each chimera was tested for the ability to rescue depletion of endogenous EphA4 in the mesoderm or EphB4 in the ectoderm. Depleted Ephs are represented by “ghost” shapes. Separation could be rescued by the constructs that contained the corresponding extracellular domain (AB for EphA4, BA for EphB4), but not by the constructs that contained the cytoplasmic domain. A* and B* were control constructs, which were wild type except for two amino acids within the end of the transmembrane domain, which had to be substituted in AB and BA to produce the chimeric constructs (see Materials and Methods).

To address the issue of repulsion restriction, we conducted a comprehensive characterization of the ephrin–Eph system in the early gastrula. We first compared quantitatively the transcripts of all ephrins and Ephs in the dorsal ectoderm and in the mesoderm. Both tissues expressed multiple ephrins and Ephs. Although both A and B types are involved at the ectoderm–mesoderm boundary ([5] and unpublished data), the contribution of the A type appears less important, as they are not sufficient on their own to induce separation ([4] and unpublished data). We thus focused on the ephrin B subfamily and their receptors, which showed predominantly asymmetric patterns (Figure S1B). Thus, ephrinB2 and EphA4 were strongly enriched in the mesoderm, whereas the ectoderm selectively accumulated ephrinB3 and EphB2–4. We next wanted to test whether asymmetric expression is functionally relevant for tissue separation. Interestingly, ephrinB2, ephrinB3, and EphA4 start to be expressed just at the onset of gastrulation (Figure S1A), coinciding with the appearance of the ectoderm–mesoderm boundary and the onset of separation behavior [19]. We thus focused on ephrinB2, ephrinB3, EphA4, EphB2, and EphB4 as strongly asymmetrically distributed molecules and included in our analysis ephrinB1 as an example of an evenly expressed ligand (Figure 1C).

Although ephrin/Eph depletion severely disrupts the endogenous ectoderm–mesoderm boundary ([4] and Figure 1A), the embryonic phenotype is difficult to interpret, due to multiple functions of ephrins and Ephs in various aspects of gastrulation [5],[20]–[22] (Winklbauer unpublished). We thus performed most of our study on a reconstituted boundary produced by apposition of ectoderm and mesoderm explants (Figure 1B,B′), an assay that allows an in-depth dissection of tissue separation [4],[19],[23]. By systematic depletions using antisense morpholino oligonucleotides (MOs) (Figure S2A,D), we established that each ephrin and each Eph is required, either in the ectoderm, in the mesoderm, or in both. Their depletion inhibited tissue separation to a degree that generally correlated with their relative tissue enrichment (Figures S1B and S2A). Interference with ephrins or with Ephs on one side of the boundary, by single or multiple depletions (Figure S2A) or dominant negative constructs [4], led to a maximal reduction of separation to 30–40%, whereas simultaneous interference on both sides of the boundary led to a significantly stronger inhibition (Figure S2A), consistent with a requirement for two antiparallel forward signals [4].

After having established that each ephrin and Eph subtype is required, we asked next whether a given subtype could be replaced by another member of the family. An ephrin or Eph was depleted, and rescue was attempted by mRNA injection (Figure S2B), or by direct activation at the boundary through incubation with soluble preclustered ephrin or Eph extracellular domains (Figure 1D). Results from both kinds of rescues were in perfect agreement. Subtypes typically failed to substitute for each other, as observed for ephrinB1 and B3, or for EphA4 and B4. The only exception was ephrinB2, which could substitute for ephrinB3 (Figure 1D), a result that will be explained in later experiments. The apparent lack of rescue was not due to a lower activity of a particular construct or Fc-fragment, as rescue of the same subtype was in all cases efficient. This was confirmed by comparing two different amounts of ephrinB1 and B3 mRNAs (Figure S2B) and verifying their expression levels (Figure S2C). The lower amount was sufficient to rescue depletion of the same subtype, but depletion of the other subtype could only be marginally rescued with the highest amount. These results demonstrated an unexpectedly tight, although not absolute, specificity of the requirements for each ephrin and Eph. Note that the ability of the EphA4-Fc fragment to rescue loss of EphA4 also uncovered a contribution from reverse signaling to tissue separation.

### The Specificity of Eph Receptors Resides in Their Extracellular Domain

The specific requirement for all ephrinB subtypes and their receptors could be due to specific differences in downstream signaling and hence to different roles during tissue separation. Alternatively, signaling could be uniform and additive, but depend on specific receptor–ligand interactions. To determine whether Eph receptor specificity resided in their cytoplasmic tail or in their extracellular domains, we constructed EphA4/B4 chimeras, where the respective cytoplasmic domains were swapped, which we tested for the ability to substitute for endogenous EphA4 or EphB4 proteins. We verified that these chimeric constructs were properly expressed at the cell surface (Figure S3A) and activated by ephrin-Fc fragments (Figure S3B). The functional assays gave clear-cut results (Figure 1E): The AB chimeric construct, which contained the extracellular domain of EphA4 and the intracellular domain of EphB4, could perfectly rescue the loss of EphA4, but was unable to substitute for EphB4. Reciprocally, the BA construct could rescue EphB4 but not EphA4 depletion. These results showed that the extracellular domains were responsible for the Eph specificity. The cytoplasmic domains appeared interchangeable, suggesting that the requirement for each of these Eph receptors was probably not due to differences in signaling but rather reflected the ability to bind selected ligands. We postulated that specific combinations of receptor–ligand pairs could underlie the restriction of repulsion to the boundary.

### Complementarily Expressed Ephrins and Ephs Selectively Interact to Form Functional Pairs

**Figure 2:**
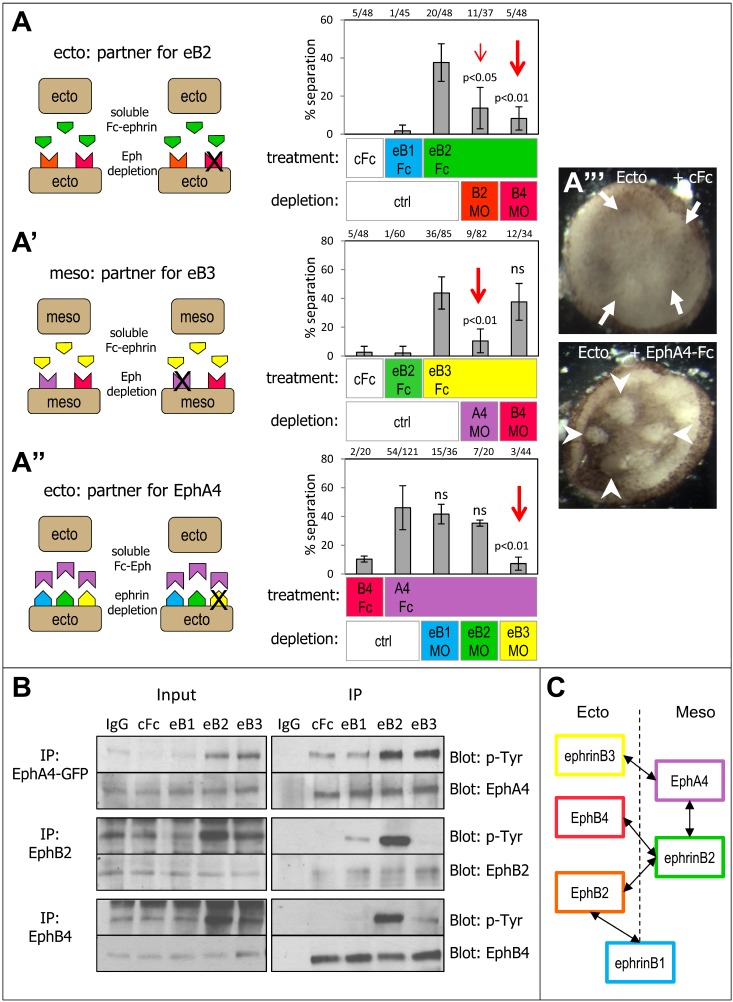
Characterization of ephrin–Eph specificity. (A) Identification of functional cognate receptors. The in vitro separation assay was performed using two explants from the same tissue (ectoderm–ectoderm or mesoderm–mesoderm). Under normal conditions these explants mix completely. Separation could be induced when these explants were exposed to soluble Fc fragments corresponding to ephrins or Ephs normally enriched in the other tissue (see Figure 1C), thus mimicking the endogenous asymmetric ephrin/Eph expression. The endogenous functional partners were then identified by depleting single candidate ephrins/Ephs in the receiving explant and determining which of them was required for ectopic separation. Note that in this set of experiments, Fc fragments were present during the assay, ensuring that both explants were continuously exposed. Separation can also be induced by treating only one explant, although the penetrance is lower [4]. (A) Separation of ectoderm explants was induced by soluble Fc fragments corresponding to “mesoderm-specific” ephrinB2, but not ephrinB1, already endogenously enriched in the ectoderm. EphrinB2-Fc–induced separation was strongly inhibited by EphB4MO and more weakly by EphB2MO. (A′) Mesoderm aggregates were tested on large mesoderm layers artificially produced in the animal pole of the embryo (see Materials and Methods). Separation was induced by Fc fragments of “ectoderm-specific” ephrinB3, but not ephrinB2. Ephrin3-induced separation was reversed by EphA4MO but not by EphB4MO (red arrow). (A″) Ectopic separation between ectoderm explants was induced specifically by “mesodermal” EphA4 but not by “ectodermal” EphB4. Separation was inhibited only by ephrinB3MO (red arrow). (A″′) Examples of mixing of ectoderm explants incubated with control Fc (arrows) and of separation of ectoderm explants treated with EphA4-Fc (arrowheads). (B) Biochemical analysis of differential activation of Eph receptors by ephrin ligands. Ectoderm explants were treated with 40 nM ephrinB1, B2, B3 Fc fragments or with control anti-Fc antibodies for 1 h, and then lysed. Endogenous EphB2 and EphB4, and EphA4 were immunoprecipitated and analyzed by immunoblotting for phospho-tyrosine and for total Eph levels. In the case of EphA4, which is only expressed at low levels in the ectoderm, EphA4-GFP was ectopically expressed. EphA4 was strongly phosphorylated in response to both ephrinB2 and ephrinB3, but not ephrinB1, which gave levels similar to negative controls. EphB2 responded strongly to ephrinB2, and only weakly to ephrinB1. EphB4 was highly phosphorylated in response to ephrinB2, whereas activation by ephrinB1 or ephrinB3 was negligible. IgG, control immunoprecipitation with nonimmune IgGs. (C) Summary of the main ectoderm and mesoderm-enriched ephrins/Ephs and of the preferred functional interactions.

If indeed specific ephrin–Eph pairs formed preferentially at the boundary, we predicted that ectopic addition of ephrins normally enriched in the mesoderm should induce artificial separation of two ectoderm explants. This prediction was fully verified. Indeed, ectoderm explants could be induced to repel each other by treatment with soluble Fc fragments for mesoderm-enriched ephrinB2 but not ephrinB1 (Figure 2A). Similarly, the separation of two mesoderm explants was efficiently induced by incubation with ephrinB3, which is normally expressed only in the ectoderm, but not by ephrinB2 (Figure 2A′). Ectoderm–ectoderm separation could also be induced by “mesodermal” EphA4-Fc, but not by EphB4-Fc (Figure 2A″ and A″′).

The sensitivity of ectoderm and mesoderm to respond to a subset of ephrin/Eph fragments implied that each of these two tissues must specifically express respective partners. We used the induction of ectopic separation as a functional assay to identify these endogenous receptors. Explants depleted of single ephrins or Ephs were tested for their ability to separate upon incubation with an Fc fragment. Among potential candidate receptors for ephrinB2 in the ectoderm, EphB4 depletion strongly inhibited ephrinB2-Fc–induced separation, whereas EphB2 depletion had a weaker effect (Figure 2A). The ability of ephrinB3 to induce mesoderm separation was entirely dependent on the presence of EphA4 (Figure 2A′). Finally, of all the three ephrin ligands expressed in the ectoderm, ephrinB3 was clearly the one responsible for EphA4-Fc–induced separation (Figure 2A″). Although some of these results were consistent with the relative mRNA enrichments of the various ligands/receptors (Figure S1B), others clearly implied functional selectivity. For instance, in EphA4-induced ectoderm–ectoderm separation, an explanation based only on relative expression levels and under promiscuous binding could explain the minimal role of ephrinB2, which is scarce in the ectoderm, compared to abundantly expressed ephrinB3. However, this explanation would predict a much stronger effect of ephrinB1 depletion, as the latter is present at significant levels. Thus, the best explanation was that ephrinB3 and EphA4 tended to interact preferentially.

We directly assessed functional selectivity at the level of Eph activation (Figure 2B). After treatment of ectoderm explants with equal concentrations of ephrinB1, B2, or B3 Fc fragments, each Eph receptor was immunoprecipitated, and the levels of phosphorylation were monitored by Western blot using an anti–phospho-tyrosine antibody [11]. Because of poor immunoprecipitation of the endogenous EphA4 protein with available antibodies, EphA4-YFP was ectopically expressed and immunoprecipitated with an anti-GFP antibody. In these experiments, cells were stimulated for 30 min, a time that should be sufficient to reach a bona fide steady state in terms of repulsive behavior, as separation can be induced already after a few minutes [4]. The results were clear cut: EphA4 was highly phosphorylated in response to ephrinB2 or ephrinB3 but not ephrinB1. EphB2 responded strongly to ephrinB2 and weakly to ephrinB1. EphB4 was activated by ephrinB2 but neither ephrinB1 nor ephrinB3. These results correlate well with ephrin–Eph affinities measured in vitro [13], with the notable exception of ephrinB1–EphB2, for which the affinity was reported to be similar to ephrinB2–EphB2 [24]. Combined with the asymmetric distribution of the various molecules, the differences in binding largely explained our functional data. For instance, the failure of EphB4 to induce ectoderm separation was due to the low levels of ephrinB2 in this tissue, which is its only strong interactor. On the other hand, the ectoderm could respond to EphA4 via ephrinB3, but not ephrinB1.

Taken together, our results show that each Eph receptor is selective for one or at most two ephrins. These results support the hypothesis that tissue separation is driven by asymmetric expression of specific receptor–ligand pairs. Most of the ephrin/Eph pairs identified functionally show indeed partially complementary expression (Figure 2C and Figure S1B). This is clearly the case for ectodermal ephrinB3 and mesodermaly enriched EphA4. Likewise, ectodermaly enriched EphB2 and EphB4 interact best with mesodermaly enriched ephrinB2. However, not all factors are expressed in simple complementary patterns. In particular, EphA4 interacts not only with ectodermal ephrinB3 but equally well with ephrinB2, which is abundant in the mesoderm. In contrast, ephrinB1, evenly expressed in both tissues, can only weakly activate EphB2, which is enriched in the ectoderm. Note that ephrinB1 depletion in either tissue had a rather strong phenotype (Figure 1D and Figure 2A) considering that it seemed to only interact weakly with EphB2. This may be indicative of the occurrence of an additional receptor for ephrinB1 yet to be identified (see also supplementary discussion in Text S1).

### Complementary Expression of Specific Ephrin–Eph Pairs Is Sufficient to Account for Tissue Separation

**Figure 3:**
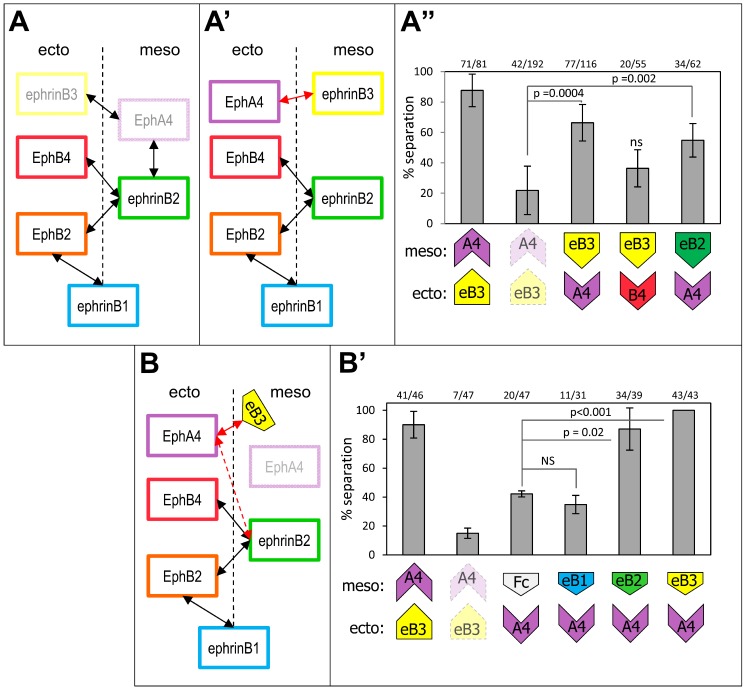
Ectoderm–mesoderm separation relies on asymmetric expression of specific ephrin/Eph pairs across the boundary, irrespective of the direction of the asymmetry. (A, A′, A″) Reciprocal replacement of mesodermal ephrinB3 and ectodermal EphA (mRNA injection). (A, A′) Diagram describing the experiment. (A) Endogenous ephrinB3 and EphA4 were depleted in the mesoderm and ectoderm, respectively (ghost labels). (A′) EphrinB3 (or B2) was then overexpressed in the ectoderm and EphA4 (or B4) in the mesoderm, thus effectively swapping the ligand and receptor (red double arrow). (A″) Quantification. Swapping ephrinB3 and EphA4 efficiently restored separation. EphrinB3 could be replaced by ephrinB2, consistent with the latter also being a ligand for EphA4. However, EphA4 could not be substituted by EphB4, in agreement with EphA4 being the only receptor of ephrinB3. The weak nonsignificant rescue was likely due to a slight boost in the ephrinB2–EphB4 signal. (B, B′) Similar experiment, but with the ephrin ligand substituted by direct incubation of the receiving explant with the corresponding soluble Fc fragment. EphrinB3 and EphA4 were depleted, EphA4 was overexpressed in the ectoderm, and ectoderm explants were incubated with the indicated Fc fragments. Overexpression of EphA4 led to a partial rescue of separation (control Fc), likely by activating ephrinB2–EphA4 signaling across the boundary (red dashed double arrow). Incubation with ephrinB3-Fc fully rescued separation. EphrinB2-Fc but not the ephrinB1-Fc fragment could also rescue, in agreement with the selectivity of EphA4.

Our results suggested that tissue separation may be simply explained by the complementary expression of selective ephrin/Eph pairs, which would generate an excess of repulsive signal at the boundary (Figure 3A). Nevertheless, it is conceivable that particular ephrins and Ephs would also be specifically needed on one or the other side of the boundary. We examined this possibility for the ephrinB3–EphA4 pair, which is closest to a fully complementary expression pattern. We depleted ephrinB3 in the ectoderm and EphA4 in the mesoderm and asked whether separation could be rescued by ectopic re-expression of these two molecules in the opposite tissues. The results were unambiguous: Swapping ephrinB3 and EphA4 efficiently restored separation (Figure 3A′). The same result was obtained when ephrinB3 was provided as a soluble ligand to the surface of the ectoderm (Figure 3B′). Thus, the presence of ephrinB3 and EphA4, respectively, in the ectoderm and the mesoderm is not required for separation; the complementary expression appears sufficient.

We tested the specificity of the process by attempting the rescue with other ephrins or Ephs. EphrinB2, which is another good partner for EphA4, could substitute for ephrinB3 (Figure 3A′,B′). No rescue was observed with ephrinB1, which only activates EphB2, nor with EphB4, which cannot function as an ephrinB3 receptor (Figure 3A″,B′).

### Separation Requires the Kinase Activity of Eph Receptors

Although EphA4 and B4 cytoplasmic tails appear interchangeable (Figure 1E), we also know that this tail is required for Eph function in separation [4]. We thus asked whether the kinase activity was involved by using kinase dead (KD) variants, where the ATP binding site was mutated [25],[26]. We found that both EphA4KD and EphB4KD failed to rescue depletion of the corresponding endogenous receptors (Figure S4A). On the contrary, they acted as dominant negatives, decreasing levels of receptor phosphorylation (Figure S4D) and inhibiting separation (Figure S4A) to similar levels as MO depletion or expression of cytoplasmic truncated forms (Figure S2 and [4]). This inhibition was fully rescued by co-expression of wild-type EphA4/B4 (Figure S4A). We further tested the effect of these KD mutants in two other situations—that is, (a) in ectopic separation of two ectoderm, or of two mesoderm explants by soluble ephrinB2, respectively, B3 Fc fragments (as in Figure 2A), or (b) in rescues of ephrinB2/B3 depletions by the corresponding Fc fragments (as in Figure 1D). In both types of experiments, expression of the EphA4KD blocked the action of ephrinB3 Fc, and EphB4KD that of ephrinB2 (Figure S4C). Thus, these KD forms behaved in all cases as strong dominant-negative mutants, demonstrating that their kinase activity is essential for ectoderm–mesoderm separation.

### Enhanced Eph Signaling and Myosin Activation at the Ectoderm–Mesoderm Boundary

**Figure 4:**
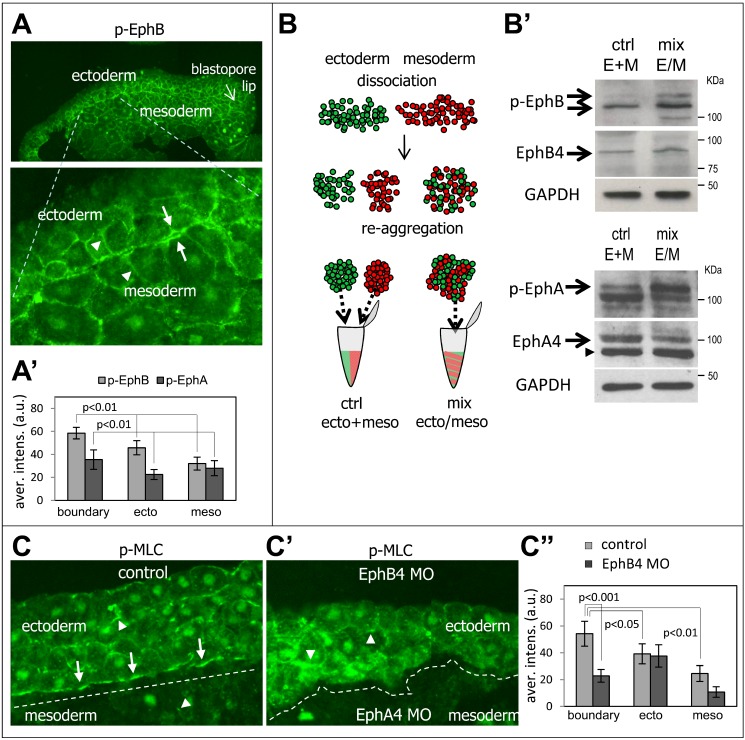
Increased Eph receptor and myosin activation at the ectoderm–mesoderm contacts. (A) Detection of phospho-EphB by immunofluorescence of a sagittal section from wild-type early gastrula embryo. The upper panel shows a general view of the dorsal region, and the lower panel an enlarged view of the ectoderm–mesoderm boundary. (A′) Quantification of relative signal intensity (arbitrary units) measured for phospho-EphB and phospho-EphA at cell–cell contacts along the boundary and inside each tissue. Averages from five embryos for phospho-EphA and nine embryos for phospho-EphB. *p* values = Student *t* test. (B, B′) Biochemical comparison of Eph phosphorylation levels between homogenous tissue aggregates and mixed ectoderm mesoderm aggregates. (B) Schematic description of the experiment. Dissociated ectoderm and mesoderm cells were mixed and left to reaggregate for 30 min, which produced a maximal number of “heterotypic” ectoderm–mesoderm contacts mimicking contacts at the boundary. Homogenates from these mixed aggregates (E/M mix) were compared to the same amount of cells assembled into separate ectoderm and mesoderm aggregates, thus forming only “homotypic” contacts, and combined during homogeneization (E+M ctrl). (B′) Western blots were probed for total and phospho-EphA, phospho-EphB, total EphA, and EphB. Mixed aggregates showed a reproducibly higher level of p-Eph signals (arrows), which indicates stronger activation at boundary contacts. Arrowheads, nonspecific bands. GAPDH was used as loading control. (C, C′, C″) Selective accumulation of p-MLC along the ectoderm–mesoderm boundary and its dependence on ephrin/Eph signaling. Ectoderm and mesoderm explants were combined, incubated for 1 h, and fixed. Cryosections were immunostained for p-MLC. (C) Normal boundary (underlined by a dashed line) between wild-type tissues. p-MLC levels are higher in the ectoderm than the mesoderm, but highest along the boundary (arrows). Arrowheads point to p-MLC signal along membranes within each tissue. (C′) Loss of p-MLC staining at the tissue interface upon Eph depletion: EphB4-depleted ectoderm and EphA4-depleted mesoderm showed largely unchanged tissue staining, but p-MLC was prominently missing from the fused interface, delineated by the dashed line. (C″) Quantification of p-MLC signal intensity at cell–cell contacts at the boundary and inside the tissues in control and Eph morpholino conditions. Average from nine embryos. *p* values = Student *t* test.

Immunostaining of sections from wild-type gastrulae with an antibody recognizing a conserved phosphorylated site present in all EphBs demonstrated that Eph signaling was indeed activated in both ectoderm and mesoderm, but was significantly stronger at the boundary (Figure 4A,A′). A similarly increased signal was observed with an anti–phospho-EphA antibody (Figure 4A′). We confirmed biochemically the existence of basal signaling in the tissues and enhanced activity at ectoderm–mesoderm contacts. Such contacts were maximized by mixing dissociated ectoderm and mesoderm cells to produce heterogeneous aggregates (Figure 4B). The levels of phosphorylated EphAs and EphBs in extracts of mixed aggregates were compared with those of pure ectoderm and mesoderm aggregates. Mixed aggregates showed higher p-EphA and p-EphB signals than combined homogenous ectoderm and mesoderm aggregates. We further showed that EphA phosphorylation in these ectoderm–mesoderm aggregates required ephrinB3, but not ephrinB1, further confirming the specificity of EphA4 (Figure 2D′). Thus, high local Eph activation is consistent with cell repulsion being restricted to the boundary, due to the preferential interactions between complementary pairs of ephrins and Ephs enriched on opposite sides of the boundary.

The typical mechanical output of Ephrin/Eph signaling in repulsive behavior involves myosin-based contraction [27],[28]. We determined the distribution of phosphorylated myosin light chain (p-MLC) in the ectoderm and mesoderm tissues and at their interface (Figure 4C,C″). We observed p-MLC at cell–cell contacts within each tissue (arrowheads). The signal was significantly stronger in the ectoderm than in the mesoderm, but by far the most intense signal was consistently found in patches along the boundary (arrows), as expected from strong bursts of ephrin/Eph signaling at this interface. EphA4/EphB4 depletion strongly decreased p-MLC staining at the boundary and in the mesoderm, but not in the ectoderm (Figure 4C′,C″). These results confirm that the boundary is a site of significantly high contractile activity that largely depends on Eph signaling.

### Separation Is Controlled by the Balance Between Ephrin–Eph-Dependent Repulsion and Cadherin-Mediated Adhesion

Significant Ephrin–Eph signaling also takes place within each of the two tissues (Figure 3A,B). To account for the fact that the tissues remained coherent and overt cell detachments occurred only at the interface, we considered the role of cell–cell adhesion. We proposed that cohesion or separation is determined by a balance between cadherin adhesion and Eph signaling-dependent contractility. Although cadherin levels are lower in the most anterior mesendoderm ([29]–[31] and unpublished data), they are quite similar between the ectoderm and the mesoderm analyzed in our experiments (Figure S5B), implying that the balance would mostly depend on the strength of Eph signaling. This signaling would take place at all cell contacts, but only at the boundary would the signal be sufficiently intense to overcome adhesion, thus causing cell detachment.

This model predicted that increasing cadherin levels or decreasing contractility should inhibit detachments between ectoderm and mesoderm cells, while on the contrary decreasing cell adhesion or increasing Eph signaling should lead to visible detachments between mesoderm cells. This hypothesis was consistent with the observation that ectoderm–mesoderm separation was impaired upon cadherin overexpression, which could be rescued by boosting Eph signaling across the tissue interface by incubation with soluble ephrin Fc fragments (Figure S5A). Note that Eph depletion did not significantly affect cadherin levels (Figure S5B), arguing against a direct regulation of cadherins by ephrin–Eph signaling.

**Figure 5:**
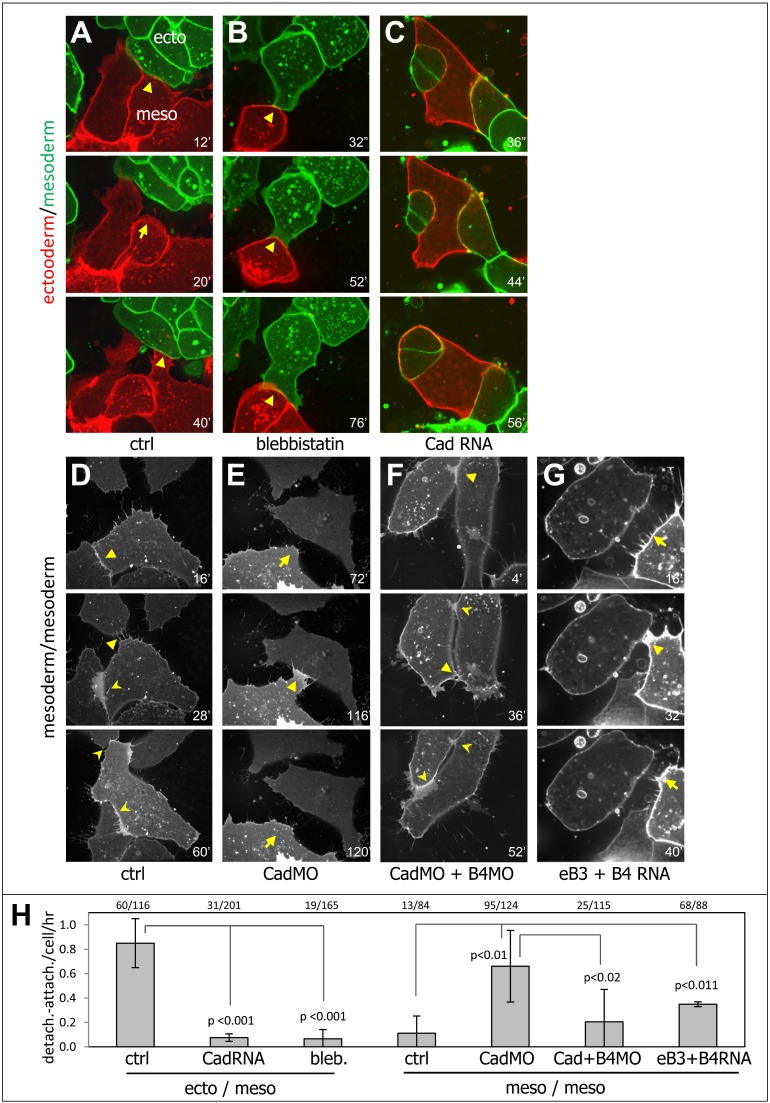
Tissue separation is controlled by a balance between ephrin/Eph-mediated repulsion and cadherin adhesion. (A–C) Ectoderm–mesoderm repulsion requires myosin activity and is antagonized by cadherin adhesion. Selected frames from time lapse confocal movies (Materials and Methods) showing dynamics of cell–cell contacts between single embryonic cells. Cells dissociated from ectoderm and mesoderm tissues were plated on glass coated with low amounts of fibronectin (see Materials and Methods). Ectoderm cells expressed membrane-GFP, and mesoderm cells membrane-Cherry. (A) Wild-type ectoderm and mesoderm cells stably attached to cells from the same tissue, but contacts between ectoderm and mesoderm cells exhibited cycles of attachments (arrowheads) and detachments (arrows) recapitulating the separation behavior observed at the boundary between the two tissues. (B) After treatment with 100 µM blebbistatin, most ectoderm mesoderm contacts remained stable. (C) C-cadherin overexpression in both ectoderm and mesoderm strongly decreased detachments. Note that mesoderm cells tended then to surround ectoderm cells. (D–G) Evidence for subthreshold levels of Ephrin/Eph-mediated repulsive signals between mesoderm cells. (D) Mesoderm cells (here control MO-injected) established stable contacts (arrowheads) that were maintained (concave arrowheads) throughout the duration of the recording (1 h). (E) Cadherin-depleted mesoderm cells (cadherin MO) showed frequent figures of redetachments (arrows), indicating the existence of repulsive signals. (F) Stable contacts between cadherin-depleted mesoderm cells were rescued by simultaneous Eph depletion, demonstrating that the repulsion observed between mesoderm cells was due to ephrin–Eph signaling. (G) Detachment between mesoderm cells could be induced by increased ephrin–Eph-mediated repulsion through ectopic expression of ephrinB3 and EphB4, the “preferred” ectoderm partners for “mesodermal” EphA4 and ephrinB2. Frame numbers of the corresponding movies are indicated. (H) Quantification of the rate of attachment/detachment per cell per hour. Numbers indicate number of events per number of cells analyzed.

To explore the behavior at individual homotypic and heterotypic cell–cell contacts, we juxtaposed single cells obtained by dissociation of early gastrula tissues (Figure 5A–I and Movies S1–S7). Our dissociation conditions fully preserved both the capacity of cells of the same tissue to rapidly re-establish stable adhesions (Figure 5D–G) and the ability for contacts between ectoderm and mesoderm cells to reproduce the alternating cycles of attachment/detachment characteristic of the separation behavior (Figure 5A–B and Movie S1). We first tested the effect of increasing cadherin levels on the normal repulsion between ectoderm and mesoderm cells. We also tested the effect of incubating the cells with the myosin inhibitor blebbistatin. Both conditions severely reduced the frequency of detachments (Figure 5B,C,H and Movies S2 and S3), consistent with the inhibition of tissue separation observed under the same manipulations (Figure S5A).

Because we expected mild repulsion between mesoderm cells, we predicted that detachments should become more prominent if adhesion would be experimentally decreased. We subjected mesoderm cells to a mild cadherin depletion (∼30%, not shown) and observed that contacts were now much less stable and cells displayed the typical repulsive behavior normally observed at contacts with ectoderm cells (Figure 5E,H and Movies S4 and S5). These retractions were entirely dependent on intact ephrin/Eph signaling (Figure 5F,H and Movie S6). We also predicted that mesoderm cells could become repellent even with normal cadherin levels if repulsive signals would be increased. We chose to express ephrinB3 and EphB4, because they are normally enriched in the ectoderm, and are the respective specific partners for mesoderm-enriched EphA4 and ephrinB2 (Figure 2C). EphrinB3/EphB4-expressing mesoderm cells readily showed strong repulsion (Figure 5G,H and Movie S7). This balance between repulsion forces and cell–cell adhesion was also observed at the tissue level in reaggregation assays (Figure S5C–E), in which diminished cohesion induced by cadherin depletion was rescued by co-depletion of EphB4, whereas ectopic expression of ephrinB3 and EphB4 decreased cohesion of cells with wild-type cadherin levels.

### Simulating the Ephrin/Eph Network Reveals the Robustness and the Limits of the Model

Our results suggested that separation could be explained by the asymmetric expression of a subset of specific ephrin–Eph pairs, which resulted in widespread ephrin–Eph signaling, but with an altogether higher output across the boundary. Although the concept is intuitively coherent, the actual system is complex, with many ephrins and Ephs, including pairs that could interact extensively within one tissue (e.g., ephrinB2–EphA4 in the mesoderm). To better estimate the validity of this model, we simulated it by computing the contribution of all the various ephrin–Eph interactions established at homotypic contacts within the tissues and heterotypic contacts between tissues (Figure S7). We considered a minimal model taking into account two parameters: (a) the affinities between the extracellular domains of ephrins and Ephs and (b) their relative expression in the different tissues (Text S1 and Figure S7). Affinities for the various ephrin–Eph pairs have been measured in vitro [13]. These values remain imprecise, but were globally consistent with our data, with few exceptions (e.g., ephrinB1–EphB2; see Text S1). Endogenous levels for the different ephrin and Eph proteins could not be measured directly, due to lack of adequate antibodies, but were approximated based on relative mRNA levels determined by real-time PCR (Figure S1B) and on a global estimate obtained indirectly (see Text S1 and Figure S6). Note that these “apparent” affinities and concentrations are here purely operational terms. The actual system is hugely complex, constituted of multiple ligands and receptors, all of them membrane proteins, capable of spontaneous or ligand-induced clustering, and potentially also of cis-interactions [15],[32]–[37]. Most of these additional parameters and their impact on ephrin–Eph function are still ill-defined, and a formal description of such a system is a largely unresolved problem.

However, this simple model where receptor activation depends on its own concentration, the concentrations of its various potential ligands, and the corresponding affinities turned out to describe with surprising accuracy the situation at this boundary. The signal output turned out indeed to be clearly highest at the tissue boundary compared to ectoderm–ectoderm and mesoderm–mesoderm contacts (Figure S7D). The system appeared very robust, predicting a highest signal at the boundary over a broad range of ephrin–Eph apparent concentrations and of apparent KDs (Figure S7D). Quite strikingly, the result of the simulation closely resembled the relative levels of phosphorylated receptors in our immunofluorescence images (Figure S7D and E, reproduced from Figure 4A). The model was further tested for the ability to predict the outcome of loss- and gain-of-function experiments (Figure S7F–J). The simulation recapitulated very well the major characteristics of the system. In particular, the specific induction of “separation” between ectoderm cells by activation with mesoderm-enriched ephrinB2 or EphA4, the inhibition of normal separation upon ephrin or Eph depletions (Figure S7G,H), and most importantly the fact that rescue could not be obtained indifferently by any ephrin (or Eph) (Figure S7H) argued against widespread ligand–receptor promiscuity. It also accounted for our original observation that strong inhibition of separation required interfering with receptors on both sides of the boundary (Figure S7J) (see [4] and Figure S2A).

We conclude that a system of partially complementary and semiselective ephrin–Eph pairs is indeed sufficient to explain ectoderm–mesoderm separation. The robustness of the simulation provides strong support for the general principles of this model. Although multiple additional factors are likely to impact on the apparent concentrations and affinities, on the response curves of Eph activation, and more downstream on the contractile activity of boundary cells, such factors may not affect significantly the final global pattern. The reason for this robustness appears to be the very limited set of pairs that can effectively interact and thus influence the system, and the fact that three out of five of these pairs are asymmetrically expressed (ephrinB3–EphA4, ephrinB2–EphB2, and ephrinB2–B4). The simulation indicated that, for these five pairs, differences up to 10-fold in apparent affinities would change the relative strength of the outputs, but the signal remained strongest at the boundary under most conditions. The few aspects of our experimental data that were not well simulated are discussed in Text S1. Discrepancies were certainly expected considering the many additional mechanisms that can modulate ephrin–Eph signaling. Nevertheless, the predictions of the simulation were surprisingly good given the simplicity of the assumptions. We conclude that a combination of multiple semiselective pairs is sufficient to explain a large part of the system's behavior.

### Selective Ephrin–Eph Pairs at the Ventral Ectoderm–Mesoderm and at the Notochord Boundaries

**Figure 6:**
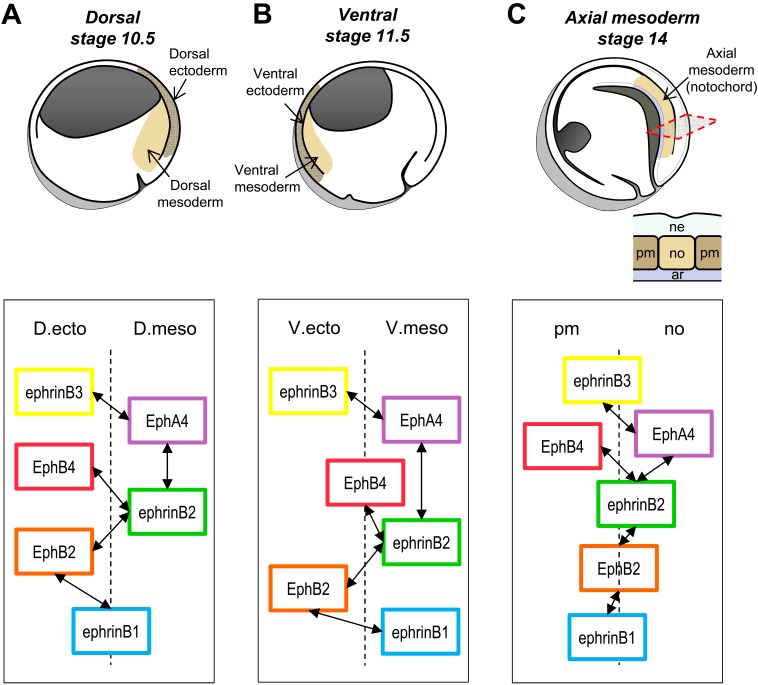
Schematic representation of the three boundaries forming during Xenopus gastrulation and simplified diagrams of the preferential ephrin–Eph pairs. (A) Dorsal ectoderm–mesoderm boundary. (B) Ventral ectoderm–mesoderm boundary. (C) Notochord–paraxial mesoderm boundary. The top drawings represent sagittal sections of the corresponding stages and highlight the two tissues forming the corresponding boundary. At stage 14, the boundaries between the notochord (no) and the paraxial mesoderm (pm) form perpendicular to the plane of the section. Cross-section is shown in insert. ar, archenteron roof; ne, neuroderm. In the lower diagrams, the position of the boxes representing each molecule symbolizes its general distribution: A box placed on one side of the boundary corresponds to strong asymmetric distribution. Weakly asymmetric or homogenous distributed molecules are drawn overlapping the boundary. The double arrows link the functional pairs. The patterns at the dorsal and the ventral boundaries are similar, with two prominent differences: EphrinB1 shifts from equal in the two dorsal tissues to more mesodermal in the ventral side. EphB4, on the contrary, shifts from mainly ectodermal to equally distributed. At the end of gastrulation, the dorsal mesoderm experiences significant changes: Compared to the earlier dorsal mesoderm, the notochord (no) keeps EphA4 and ephrinB2, but the paraxial mesoderm (pm) loses EphA4 and acquires ephrinB3 and EphB4, which were until then typically ectodermal. Note that the major asymmetries concern EphA4 and B4; the other components are only slightly enriched in one or the other sides of the boundary.

We wondered whether the principles found for the early ectoderm–mesoderm boundary would similarly apply to other boundaries. Ephrin and Eph expression is indeed widespread in the gastrula embryo, and two other important boundaries form during this phase. A ventral boundary appears at midgastrula to separate the ectoderm for the ventral mesoderm (Figure 6B), which is known to have properties different from those of dorsal axial mesoderm. At the end of gastrulation, the dorsal mesoderm is partitioned into the axial notochord and lateral paraxial mesoderm (prospective somitic mesoderm) (Figure 6C). In the latter case, we recently showed that ephrins and Ephs were indeed involved in separation [38]. We decided to systematically analyze the patterns of ephrin/Eph expression in these two regions (Figure S1B). We found that the same set of ephrins and Ephs was expressed in those regions. The expression patterns revealed common themes as well as some significant differences (summarized in the diagrams of Figure 6). In the case of the ventral boundary, the ephrinB3–EphA4 complementarity and the mesoderm enrichment of ephrinB2 were preserved. However, EphB4, which was enriched in the ectoderm on the dorsal side, was now homogenously expressed, whereas on the contrary ephrinB1, equal on both sides of the dorsal boundary, was here strongly asymmetric, now fully complementary to its receptor EphB2. In the late gastrula, the two new structures emerging from the dorsal mesoderm showed very interesting expression patterns: The notochord had preserved strong EphA4 and ephrinB2 expression, which were the major characteristics of the early mesoderm. The paraxial mesoderm, however, had dramatically modified its ephrin/Eph expression, acquiring typical features of ectoderm—that is, low EphA4 and high EphB4. Note that the complementarity was strong for Eph receptors, but rather mild for the ephrins. However, a similar trend was observed, where the notochord remained more “mesoderm-like” (higher ephrinB2, lower ephinB3), and the paraxial mesoderm was now more “ectoderm-like” (lower ephrinB2, higher ephinB3).

These observations suggested that tissues that became separated by boundaries expressed some sort of “modules” characterized in particular in all cases by complementary ephrinB3–EphA4 expression, but had some flexibility for other components. We then tested the role of key players, at both boundaries. In the case of the ventral boundary, we used the classical separation assay, attempting both loss- and gain-of-function experiments (Figure S8). All the results were in perfect agreement with the expression patterns and with our model. Similar to the dorsal boundary, depletion of either the ectoderm-enriched ephrinB3 or of its mesoderm partner EphA4 inhibited separation. In addition, ephrinB1 depletion in the mesoderm, which had only a weak effect on the dorsal side, affected here separation much more drastically (Figure S8B). Depletion in the ectoderm of its receptor EphB2 similarly inhibited separation.

In gain-of-function experiments, both mesoderm-enriched ephrinB1 and B2 could induce robust separation between two ectoderm explants when added as soluble Fc fragments (Figure S8C), an effect that could not be obtained with ephrinB1 on the dorsal side (Figure 2A). We used this phenotype to identify the functional partner of ephrinB1 on the ectoderm surface. We found that EphB2, but not EphB4, depletion significantly inhibited separation, functionally validating our biochemical results (Figure 2B).

**Figure 7:**
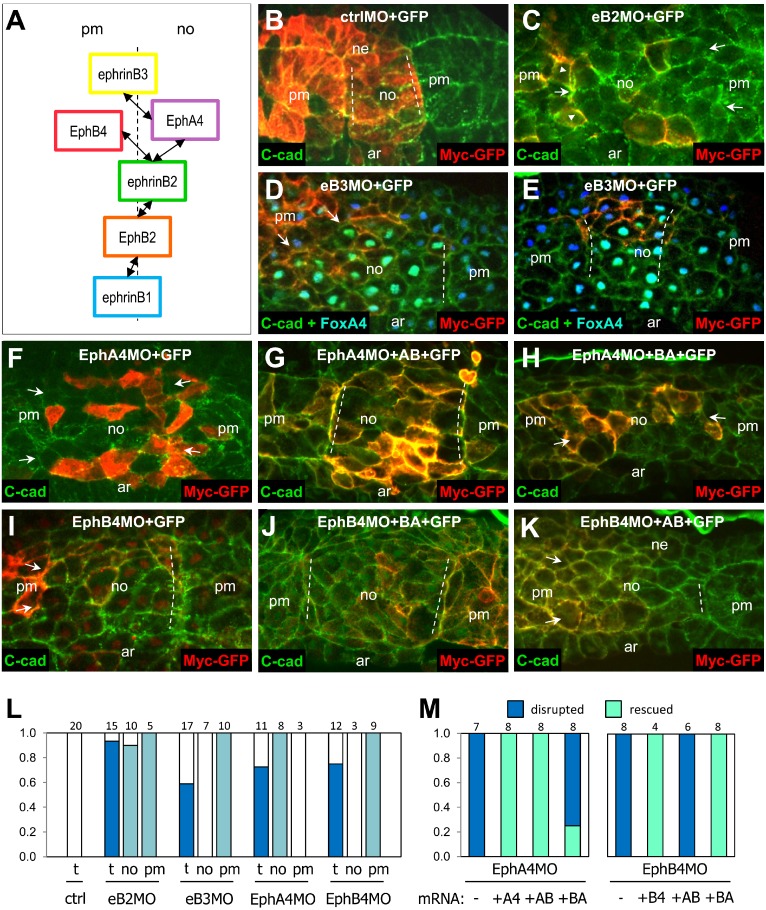
Partially complementarily expressed ephrins and Ephs control notochord–paraxial mesoderm separation. (A) Summary of ephrin and Eph expression and of the functional pairs (double arrows) in the dorsal mesoderm at stage 14 (see Figure S1B). (B–M) Manipulations were targeted to a restricted region of the dorsal mesoderm. Embryos were fixed at stage 14, and the dorsal structure was analyzed on sections. Injected cells were detected by the tracer Myc-GFP (red). Only the strongest signal is visible on these images but is sufficient to indicate the position of the injected area. Membranes were labeled with an anticadherin antibody (green). In (D) and (E), FoxA4-positive nuclei appear in green–cyan. FoxA4 is used as the notochord marker. (B) Control. Normal boundaries (highlighted by dashed lines) were characterized by a smooth alignment of the notochord and paraxial mesoderm cells. (C) Strong boundary disruption in an ephrinB2 MO-injected embryo. The position of the left boundary can still be located (arrowheads), but the alignment is jagged (arrowhead). On the right side, the two tissues are continuous, without a detectable boundary. Arrows point to the approximate limit of the notochord. (D and E) The boundary is effectively disrupted by ephrinB3 MO targeted to the paraxial mesoderm (arrows in D), but not to the notochord (E). (F–H) EphA4 MO injection in the notochord, the tissue fused with the paraxial mesoderm (F, arrows). A boundary can be rescued by the coinjection of mRNA coding for the AB Eph chimera (G, dashed lines) but not by the BA chimera (H, boundaries lack on both sides, arrows). Thus, the extracellular domain of EphA4 is necessary and sufficient for its function in the notochord. (I) Inhibition of separation by EphB4 MO targeted to the paraxial mesoderm. Left boundary is missing (arrows). (J) Rescue by the BA chimera. The injection was targeted to the right boundary. (K) The AB chimera fails to rescue. The injection was targeted to the left side (arrows). The right boundary is intact (dashed line). (L) Quantification of boundary disruption by ephrin/Eph depletion. Individual boundaries were scored as follows: 1, intact boundary; 0.5, partially disrupted boundary (rare cases); 0, fully disrupted boundary. The first column (t) compiles data of all embryos, (no) and (pm) the number of boundaries where injection was mainly targeted to the notochord or to the paraxial mesoderm. Numbers on top are numbers of embryos (2–6 independent experiments). EphrinB2, which can interact with both EphA in the notochord and EphB4 in the paraxial mesoderm, was required in both tissues. EphrinB3 and EphB4 depletion strongly disturbed the boundary when targeted to the paraxial mesoderm, but had no effect in the notochord. The opposite was observed for EphA4, consistent with the expression patterns and the selective interactions. (M) Quantification of rescues by wild type and chimera forms of EphA4 and EphB4. In all cases, complete rescue was obtained with the corresponding wild-type proteins and with the chimera containing the correct extracellular domain. The nature of the intracellular domain was indifferent. Numbers on top indicate total number of boundaries in each category.

The role of ephrins and Ephs on the notochord boundary was examined by targeted depletion in a restricted region of the embryo [38]. The effect on the integrity of the boundary was examined on sections of whole embryos (Figure 7). We tested depletion of the two strongly asymmetric Eph receptors, A4 and B4, and of their ligands ephrinB2 and B3. We observed in each case strong disruption of the boundary. However, each ephrin/Eph MO disrupted the boundary only when targeted to the expressing tissue: ephrinB3 MO and EphB4 MO in the paraxial mesoderm, EphA4 MO in the notochord. EphrinB2 MO was the only one that had an effect on both sides. These phenotypes were perfectly consistent with the distribution and partial specificity of these molecules. For the receptors, which were strongly asymmetrically expressed, MO injection obviously had an effect only when targeted to the tissue where they were enriched. For the ligands, the results were explained by their specificity. Indeed, the only receptor for ephrinB3 is EphA4, enriched in the notochord; thus, despite the poor complementarity of ephrinB3 distribution, its depletion only impaired separation when targeted to the opposite tissue (paraxial mesoderm) (Figure 7D,E,L). EphrinB2, however, can stimulate both EphA4 and EphB4 and was thus predicted to significantly contribute to generate repulsion at both sides of the boundary, explaining the widespread effect of its depletion (Figure 7L). We further verified that this functional selectivity was purely due to extracellular interactions: We used the EphA4 and B4 chimeras to rescue disruption of the notochord boundary upon EphA4 or EphB4 depletion. In both cases, rescue was only achieved by expression of the chimeric construct that harbored the proper extracellular domain (Figure 7F–K,M).

## Discussion

This study reconciles several previous puzzling observations and provides a coherent description of boundary formation based on selective repulsion between two cell populations. In particular, it explains how in tissues with widespread expression of multiple ephrin ligands and Eph receptors, cell–cell repulsion due to receptor–ligand interaction can be restricted to tissue boundaries. Situations where ephrins and Ephs are co-expressed were expected to be dauntingly complex, considering the many possible cross-regulations proposed to occur and the potentially distinct pathways that each member of these families may stimulate. However, we found that the essence of the early boundaries can be represented by a surprisingly simple model where the final output can be largely predicted based on the relative selectivity of the extracellular interactions and the abundance of the various components.

It is important to emphasize that the system cannot be simply reduced to the sum of binary inputs of individual specific pairs expressed in opposite tissues, but must rather be viewed as an integrated network made of semiselective pairs. Most of the molecules have more than one partner (ephrinB2 interacts will all receptors), and expression patterns range from equal distribution on both sides of the boundary to strong enrichment in one tissue, with intermediate partial asymmetries being most frequent. These characteristics of the network explain well its reaction to experimental manipulations: Although each component was required to a degree that generally corresponded to its expression levels and distribution, the specificity of the requirement was not absolute, as it could in some cases be substituted by another subtype that shared the same partner (ephrinB2 and ephrinB3 with EphA4; Figure 1D). In other words, the role of each of the ephrins and of the Eph receptors is dictated by the possibility to establish an interaction with a partner across the boundary. Note that some weaker degree of rescue was also achieved by components that did not interact with the same partner, as in the cases of ephrinB1 and ephrinB3 (Figure S2B). In those cases, overexpression could apparently boost signaling through a different ephrin–Eph pair, although this did not efficiently compensate for the loss of the original signal (Figure S2B). These partial compensations are fully expected in this type of network. Arguably the most definitive validation of the model came from the ephrinB3–EphA4 swapping experiment (Figure 3), which demonstrated that separation did not depend on the presence of a particular ephrin or Eph in the ectoderm or the mesoderm, but on the ability of selective pairs to establish interactions across the boundary.

Having said that the system is an integrated network, it is equally important to highlight the fact that only a subset of potential ephrin–Eph combinations (five out of nine) can establish interactions of functional significance (Figure 7). This subset of functional pairs is identical to the one determined as capable of in vitro binding [13]. Thus, our data represent a solid in vivo validation of the notion of partial ephrin–Eph specificity of ligand-receptor interactions. As discussed in Text S1, the fact that ephrins and Ephs are all expressed at similar levels, that the affinities of the five functional pairs are all in the same range, and that the impact of varying these affinities is predicted to be limited allow us to summarize the system in a simple model, where all functional pairs may be considered as producing equivalent contributions. The output appears then largely imposed by the partially complementary patterns of several of these pairs (Figure 7).

Altogether, there data represent to our knowledge the most extensive dissection of network made of multiple ligands and receptors. Its success relied on the extensive use of combinations of loss- and gain-of function experiments comparing in parallel different ephrins and Ephs. Concerns often arise when attempting to evaluate the ability of one member of a gene family to substitute for another, due to the difficulty to control for the activity of the reagents. In this context, the strength of our experimental system resides in the high coherence of the systematic comparison of multiple conditions, including cross-rescues, where each reagent was validated in at least one of the complementary conditions (e.g., Figure 1D,E, and Figure S2B).

The comparison of three morphologically similar, cleft-like boundaries that form during Xenopus gastrulation and depend on Eph–ephrin signaling allowed us to extract recurrent regulatory motives in the form of complementary receptor–ligand pairs: ephrinB3—EphA4, ephrinB1–EphB2, and ephrinB2–EphB4 (Figure 7). For each boundary, at least two of these three pairs show complementary expression patterns. Moreover, we had shown previously that complete ectoderm–mesoderm boundary formation requires antiparallel ligand–receptor signaling [4]. We observed now that at all three boundaries studied here, the respective complementary pairs were arranged in an antiparallel pattern (e.g., ephrinB3–EphA4 from ectoderm to mesoderm, ephrinB2–EphB2 from mesoderm to ectoderm). Of the complementary pairs, ephrinB3–EphA4 is most systematically exploited in the different contexts, suggesting that it may be specialized in tissue separation.

Altogether, the logic of ephrin/Eph-dependent repulsion regulation at tissue interfaces can be reconstructed as follows. For the complete cleft-like separation, a minimum of two antiparallel receptor–ligand pairs is required. If both receptors and both ligands were completely specific, signaling would be completely restricted to the boundary, as the ligands and receptors co-expressed in each tissue would interact only with partners in the adjacent tissue. If interactions were completely promiscuous, signaling intensity within each tissue were similar to that across the boundary, leading to the disintegration of the whole array. If interactions were relatively specific, favoring signaling between complementarily expressed pairs, repulsion could be restricted to the boundary by a threshold mechanism where full cell–cell detachment occurs only above a certain level of signaling intensity. In this situation, additional expression of receptors and ligands, with additional strong or weak interactions, would be compatible with proper boundary formation as long as signaling remained below the threshold within each tissue, and exceeded it at the tissue interface. This third scenario is the one that we encountered in the embryo. Such a dynamic yet robust system appears perfectly suited for the complex morphogenesis of vertebrate embryos. For example, they allow combining multiple functions of ephrin/Eph signaling, in the same tissues ([39],[40] and Winklbauer unpublished).

The rules identified in this study provide a coherent logic to tissue separation. For instance, one now understands how separation between dorsal ectoderm and mesoderm is established at the onset of gastrulation [19]. The system builds on the EphB receptors, which are already expressed before gastrulation (Figure S1A). Their enrichment in the prospective ectoderm is the typical default distribution for maternal components. This intrinsic bias in expression becomes functionally relevant once the mesoderm starts expressing ephrinB2, the strongest ligand for EphB receptors, initiating a forward signal into the ectoderm. The simultaneous expression of ephrin B3 in the ectoderm and its receptor EphA4 in the mesoderm provides the antiparallel forward signal into the mesoderm, completing the requirement for full tissue separation at this boundary. In subsequent stages, this pattern is progressively modified. Complementary expression of ephrinB3 and EphA4 is still involved in all cases, but other components, including those which were maternally expressed, are now tightly regulated and change their expression pattern. This is the case for ephrinB1, which, while ubiquitously expressed and hence neutral during dorsal ectoderm–mesoderm boundary formation, becomes mesoderm-enriched to play a prominent role on the ventral side. Other pairs, such as ephrinB2–EphA4, should interact extensively within a tissue, presumably providing tissue-specific functions.

Are other boundaries controlled by a similar integrated network? Information of ephrin/Eph expression patterns in other systems is incomplete and not quantitative, but somites and most rhombomeres express more than one ephrin and/or Eph [1],[3], in patterns that are consistent with the basic principles described in our study: For instance, ephrinB3 and EphA4 are expressed in complementary patterns in several rhombomeres and are never enriched on the same side of the boundary. EphrinB2, on the contrary, is found both opposite to as well as on the same side as its receptors [3],[14],[41].

These findings reveal that the surface of embryonic cells is endowed with a rich array of receptors that upon direct contact with neighboring cells can establish very specific interactions. We show how such systems, by integrating the signals generated by all the combinations of high- and low-affinity interactions, can produce clear-cut decisions at tissue interfaces and at the same time tolerate a good degree of within-tissue signaling. The key to this behavior of the ephrin/Eph system is the balance of adhesion and repulsion and its regulated breakdown at a preset threshold for repulsion.

Although this model can largely explain tissue separation, our results do not exclude that particular ephrins or Ephs may play important additional roles. They may allow for the fine-tuning of the various signals, both at the boundary and within the tissues. EphrinB2–EphA4 constitutes an example of a pair that, at all stages, interacts extensively within a tissue and may provide tissue-specific functions. One such additional layer of regulation that remains to be investigated is suggested by our reaggregation assays, which showed that ephrinB2 or EphB4 depletion decreased cohesion of the ectoderm [4], indicating that at least under some circumstances these molecules behave as “pro-adhesive” in the ectoderm, while they are repulsive in the mesoderm and across the boundary.

We also provide here an important distinction between activities that are intrinsic to each tissue and reactions that occur specifically at the boundary. Our observation that global levels of myosin activation are much higher in the ectoderm is fully consistent with its well-known stiffness and much lower capacity for spreading on adhesive substrates and for migration [19],[29],[31],[42],[43]. However, we also detect a second p-MLC pool, which, unlike the former, is Eph-dependent, and highly concentrated at the boundary (Figure 3C). Despite a large difference in “basal” myosin activity, the two tissues cannot remain separated in the absence of Eph signaling. Previous hypotheses based on differential adhesion/tension fail to accurately describe this situation [29],[44],[45]. Our observations are, however, in full agreement with our model of “selective repulsion” controlled by potent and highly localized ephrin–Eph reactions, dominating at specific cell–cell contacts over the adhesive and tensile tissue properties. Adhesion (and cortical tension) does participate in the global equation by setting the general properties of the tissues, and we show here that separation results from the balance between adhesion and ephrin-mediated repulsion. The network of Ephrin signaling is thus set at the appropriate level to overcome adhesion along the boundary, without jeopardizing cohesion within the tissues. Experimental manipulation of cadherin levels can disrupt this balance, at least in the situation of the early ectoderm and mesoderm (Figure 5 and Figure S5). The notochord boundary is, however, an example where separation is remarkably robust, resisting to rather strong changes in cadherin levels [38],[46],[47]. How adhesion, basal tension, and ephrin signaling are differently set at difference boundaries to confer their specific characteristics will be an important question to investigate.

To explain his original discovery of cell sorting, Holtfreter had hypothesized that various cell types harbor different surface cues that he called “affinities.” The combinatorial network of ephrins and Ephs here unraveled provides a molecular basis to this concept. Ephrins and Ephs and other similar cell–cell contact-dependent cues are expressed in a wide variety of tissues, both in the embryo and in the adult, and we predict that the principles uncovered in the Xenopus gastrula may apply to a vast spectrum of cellular processes and account for the ability of cell types to distinguish between “self” and “nonself” and thus organize into multicellular structures.

## Materials and Methods

### Ethics Statement

All animal studies were approved by the McGill University Animal Care Committee (permit 4869 “Cellular mechanisms of embryonic boundary formation”) and the University of Toronto Animal Care Committee (permit 20010074 “Analysis of gastrulation movements in Xenopus”).

### Antibodies

Affinity-purified antibodies specific for EphB4 and EphB2 were generous gifts from Dr. Elena Pasquale (Burnham Medical Institute, [48]). Anti–phospho-EphB4, anti–phospho-EphA4, and anti-EphA4 antibodies were gifts from Dr. Greenberg [49]. Mouse anti-EphA4 was purchased from BD, pan anti-mouse ephrinB antibody from Zymed, mouse anti-alpha tubulin from Cell Signalling, mouse anti-GAPDH from ABi, mouse and rabbit anti-GFP from Invitrogen, affinity-purified polyclonal goat EphB4 from R&D, and mouse anti–phospho-tyrosine PY-20 HRP-conjugated from Santa Cruz.

### Recombinant Proteins

Recombinant mouse ephrinB2-Fc, ephrinB1-Fc, human ephrinB3, and mouse EphB4-Fc and EphA4-Fc (R&D Systems) comprising the extracellular domain of ephrin/Eph fused to C-terminal 6× histidin-tagged Fc region of human IgG were preclustered by 1 h incubation with anti-human Fc antibody (Jackson ImmunoResearch Laboratories) at a 1∶2 ratio in MBSH [4] before application. Anti-human Fc IgGs alone were used as control.

### Plasmids and Constructs

Membrane-targeted GFP and Cherry and Xenopus ephrinB1 and ephrinB2 were described previously [4]. Xenopus EphB4 and ephrinB3 in pCS2+ were gifts from Dr. A. Brändli [50]. Xenopus EphA4 (Pagliaccio) in pBluescript KS by DR Bob Winning [51] was a gift from Dr. T. Sargent. Chicken EphA4-GFP in peGFPN2 was a gift from Dr. A. Kania [52]. EphA4 was subcloned into the pCS2+and C-terminally fused to eYFP. Mouse EphA4 Y928F KD mutant in pCS2+ was a gift from Dr. Ira Daar [25]. Xenopus EphB4 KD mutant was constructed by substituting arginine to lysine at position 645 (ATP-binding) using the QuickChange site-directed mutagenesis kit. Alk4*, a constitutively active Activin receptor, was a gift from Dr. J. Smith [53]. β-catenin in pSP36T was a gift from Drs. P. McCrea and B.M. Gumbiner [54].

To construct Eph chimeras with swapped cytoplasmic domains, a restriction site was introduced at the end of the transmembrane domain in each original receptor (Nhe1 for EphB4 and Spe1 for EphA4) by site-directed mutagenesis (Quick Change XII, Stratagene). The two resulting mutants, called EphA4* and EphB4*, had, respectively, a change from bp1926 tgtcat to actagt and from bp 1763 ggtggt to gctagc corresponding to a V to L and V to A substitution corresponding to the last hydrophobic amino acid of the transmembrane domain amino acid 558–559 of Xenopus EphB4 and EphA4. EphA4* was subcloned into pCS2+ for consistency with EphB4. EphA4* and EphB4* in pCS2+ rescued loss of the corresponding endogenous Ephs with the same efficiency as the original A4 and B4 constructs (Figure 1E). The newly introduced Spe1 and Nhe1 sites were used to cut and exchange the fragments corresponding to the sequences of the cytoplasmic tails, yielding EphA4B4 (extracellular and transmembrane domains of A4 and cytoplasmic tail of B4) and EphB4A4 (extracellular and transmembrane domains of B4 and cytoplasmic tail of A4).

### Injections

mRNAs were synthesized according to the manufacturer's instructions (mMessage mMachine kit, Ambion). MOs and mRNAs were injected animally in the two blastomeres of two-cell stage embryos to target the ectoderm, and equatorially in the two dorsal blastomeres of four-cell stage embryos to target the mesoderm. MO sequences. The amounts of MO and of mRNA injected are listed in the Text S1.

### In Vitro Separation Assay

mRNA was injected animally at the two-cell stage for ectoderm expression and dorsally at the four-cell stage for dorsal mesoderm expression. Dissections and assays were performed in Modified Barth Solution (MBSH) containing: 88 mM NaCl, 1 mM KCl, 2.4 mM NaHCO3, 0.82 mM MgSO4, 0.33 mM Ca(NO3)2, 0.41 mM CaCl2, 10 mM Hepes, adjusted to pH 7.4 with NaOH and supplemented with 10 µg/ml streptomycin sulfate and penicillin. For the standard assay, explants were dissected at stage 10+ (early gastrula). Ectoderm or mesoderm aggregates were laid on ectoderm caps, and the degree of separation was scored as the percentage of aggregates that did not incorporate into the cap after 45–60 min incubation [31]. In some cases, animal caps were induced into mesoderm by injection of 120 pg β-catenin and 1 ng constitutively active Activin receptor mRNAs.

Separation of ventral tissue was similarly performed using stage 11 ectoderm and mesoderm from the ventral lip. For in vitro activation using soluble ephrins/Ephs, explants were either preincubated with preclustered ephrinB/Eph-Fc fragments (40 nM unless specified otherwise) in MBSH for 15 min at room temperature (e.g., Figure 1D) or the entire assay was performed in the presence of Fc fragments (e.g., Figure 2A). For the statistical analysis, results were compared using the two-tailed paired-sample Student's *t* test.

### Reaggregation Assay

Cells from dissected mesoderm and inner layer ectoderm were dissociated in alkaline calcium-free buffer (88 mM NaCl, 1 mM KCl, 10 mM NaHCO3, pH 9.3). For reaggregation, cells were transferred to agarose-coated Petri dishes containing MBSH and incubated for 1 h under mild rotation (10 rpm) on an orbital shaker. Images were taken under a dissecting microscope at a12× magnification using a Micropublished RTV3.3 camera (Qimaging) and were analyzed for object size using ImageJ software. Two parameters were measured: average object area and area/perimeter ratio. Results of six independent experiments were normalized using wild-type ectoderm or mesoderm as reference.

### Immunofluorescence

Wild-type embryos were fixed at stage 10.5, and sagittal cryosections were prepared as described [55],[56]. Sections were stained with anti–phospho-EphB antibody and Alexa488-coupled anti-rabbit IgG (Invitrogen). Images were collected with a DMIRE2 epifluorescence microscope (Leica) equipped with a 20×/0.70IMM Corr CS oil immersion objective and an ORCA-ER camera (Hamamatsu Photonics), controlled with Metamorph software.

### Live Imaging

Tissues expressing membrane-targeted Cherry or GFP as well as various mRNAs/MOs were dissociated in dissociation buffer, cells were transferred to glass-bottom petri dishes (Fluoro dish, World Precision Instruments) coated with 0.01 mg/ml Fibronectin, and cell behavior was filmed for 2 h. Cells were imaged with a WaveFX spinning disc confocal (Quorum Technologies) mounted on an automated DMI6000B Leica microscope, controlled with Volocity 3DM software (Improvision), using a 40× HCX PL APO CS, NA = 1.25 oil objective. Images were acquired every 2 to 5 min using an EM CCD 512×512 BT camera. Image processing was performed with Metamorph (Universal Imaging Corporation) and Adobe Photoshop7 software. Processing consisted of merging two to three planes from z stacks, assigning pseudo-colors, and adjusting image contrast.

### Analysis of Eph and p-Eph Levels by Western Blot

Cells from 14 ectoderm- and mesoderm-dissected explants were dissociated and an equal amount of cells were reaggregated either as mixed ectoderm/mesoderm aggregates, or as separate ectoderm and mesoderm aggregates for 1 h on agarose-coated plates in 1× MBSH. Mixed aggregates formed both homotypic ectoderm–ectoderm and mesoderm–mesoderm contacts as well as heterotypic ectoderm–mesoderm contacts, which mimicked the contacts at the boundary. The reaggregation time was set to maximize heterotypic contacts. Separate ectoderm and mesoderm aggregates, which formed only homotypic contacts, were combined for extraction, thus yielding the same amount of material as the mixed aggregates, and served as control. Extraction was performed in 1% NP40 containing buffer as previously described [46]. Extracts were probed by Western blot for EphA, EphB, p-EphA, and p-EphB.

### Immunoprecipitation

Ectoderm tissues were dissected from wild-type 40 embryos (for EphB2 and EphB4 immunoprecipiation) or from 40 embryos injected with EphA4-YFP mRNA (250 pg per blastomere) for EphA4 immunoprecipitation. Dissected tissues were treated for 40 min with an equal concentration (40 nM) of EphrinB1/B2/B3-Fc fragments, or control goat anti-human Fc. Tissues were extracted in 10 mM Hepes, 150 mM Nacl, 2 mM EDTA, 1% NP40, supplemented with protease and phosphatase inhibitors [57]. Cleared lysates were incubated for 4–5 h with rabbit anti-EphB2, anti-EphB4, or anti-GFP (for EphA4-YFP), followed by 1 h incubation with protein A-Sepharose beads (Thermo Pierce) at 4°C. The beads were washed four times with 10 mM Hepes, 150 mM NaCl, 2 mM EDTA, 1% NP40, phosphatase inhibitors+0.5% sodium deoxycholate, 0.1% SDS. Immunoprecipitates were analyzed by Western blot for phospho-Tyrosine as well as for total EphA4, EphB2, and EphB4.

### RT-PCR and qPCR

RT-PCR was performed using mRNA extracted from whole-stage 9–12 embryos. Loading was equalized by comparing levels of FGFR. Two independent experiments showed identical temporal patterns of expression. RT-qPCR was performed using mRNA extracted from ectoderm and dorsal mesoderm at stage 10.5. qPCR was carried out using CFX 96 Thermo cycler (Biorad). The PCR reactions were set up using 5 µl of RT (20 to 50 times diluted) with 5 µl of SYBR green (Biorad) ½ dilution, 5 µl of 3× PCR-MgCl_2_ buffer (Invitrogen), and 5 µl of a 6µM solution of primers. Cycling conditions were as follows: 94°C for 15 s, and 58°C for 30 s, 72.0°C for 1 min. Quantification was based on a dilution series (five fold steps) of the whole embryo RT. Relative expression levels were normalized as ratio to ODC, a ubiquitous gene with homogenous distribution in Xenopus embryos. The sequences of the primers are listed in Text S1.

## Supporting Information

Figure S1 **EphrinB1-3, EphA4, and EphB1–4 temporal expression during early Xenopus development and their relative tissue distribution.** (A) General profile of total ephrin/Eph expression. RT-PCR was performed using mRNA extracted from whole embryos of the indicated stages. EphrinB1 and EphB1–4 are maternally expressed. EphrinB2, ephrinB3, and EphA4 are exclusively zygotic, starting at the onset of gastrulation (arrow). (B) Real-time quantitative RT-PCR of dissected tissues from stage 10.5 dorsal ectoderm and mesoderm, stage 11.5 ventral ectoderm and mesoderm, and stage 14 notochord and presomitic mesoderm. Bars express distribution between the two tissues. Error bars correspond to standard deviations (two independent series of samples). Numbers below each graph correspond to relative mRNA levels (arbitrary units), directly comparing all ephrins and Eph receptors for various tissues and stages. All values were corrected based on PCR efficiency. Average efficiencies are given above as %, with standard deviation.
(TIF)

Figure S2 **Multiple Ephrin/Eph play an additive role in tissue separation across the boundary.** (A) Individual and multiple knockdowns. Single MO injections for each ephrin or Eph yielded a mixing phenotype, the penetrance of which related to the relative enrichment in each tissue (compare to Figure S1B). For instance, separation was strongly inhibited by ephrinB3 but not ephrinB2 depletion in the ectoderm, whereas ephrinB2 depletion had a strong effect in the mesoderm. Depletion of ephrinB1 gave intermediate inhibition in both tissues. The separation remaining after multiple ephrin or Eph depletions in one tissue was ∼30%–40%. Maximal inhibition could be reached in some cases by depletion of single molecules (e.g., ephrinB3 or EphB2 in the ectoderm). Depletion of Ephs on both sides led to almost complete inhibition of separation. * and ** indicate *p*<0.05 and *p*<0.01 (Student's *t* test) compared to controls (grey columns). (B) Each ephrin/Eph is specifically required and not replaceable. Individual ephrins and Eph receptors were depleted in the ectoderm or in the mesoderm, which induced inhibition of separation (white columns). Separation could be fully rescued by coinjection of mRNA (amounts indicated as pg/injection) coding for the corresponding ephrin/Eph (same colors). Only partial rescue was observed after heterotypical expression of other forms, even when expressed at high levels. * and ** indicate, respectively, *p*<0.05 and *p*<0.01 (Student's *t* test) compared to corresponding controls (white columns). “ns,” not significant. (C and D) Comparison by Western blot of ephrin levels in wild-type and manipulated ectoderm. (C) Conditions corresponding to the experiment presented in (B). Arrow points at specific ephrin band, decreasing in eB1MO. Both bands increased in ephrinB1/3 mRNA-injected embryos. Tubulin was used as the loading control. This blot is representative of three independent experiments. (D) Single and multiple ephrin depletion. Conditions are as in Figures 1D and S2A. In this blot, ephrinBs appear as multiple bands (arrows). (D′) Conditions corresponding to experiment presented in Figure 3B. p-EphA4 (arrow) was increased in mixed aggregates (mix E/M) compared to ectoderm+mesoderm aggregates (E+M). This increase was abolished by depletion of ephrinB3 (eB3MO) but not ephrinB1 (eB1MO). Arrowheads, nonspecific bands.
(TIF)

Figure S3 **Expression of EphA4/B4 chimera constructs.** (A) Immunofluorescence. Sections from ectoderm tissues expressing the AB or BA chimeras (see Figure 1 and main text) were immunolabeled using antibodies raised against the extracellular domains of EphA4 and EphB4, respectively. GFP, immunolabeled here in red, was coexpressed as a tracer. Both chimeras were well expressed at the plasma membrane. (B) Eph phosphorylation. Eph receptors appeared as major tyrosine-phosphorylated proteins in gastrula extracts, which allowed estimation of activation levels by blotting whole extract with an anti–p-Tyr antibody. Wild-type ectoderm explants or explants expressing AB or BA chimeras were incubated with ephrinB2 or ephrinB3 fragments for 30 min before extraction. Total extracts were analyzed by immunoblot using antibodies against p-Tyr and EphA4/B4 extracellular domains. EphB4 recognized a single band, but P-Tyr and EphA4 showed multiple bands. In the case of the anti-EphA4 antibody, this reflected cross-reactivity with other Eph receptors. However, comparison of controls and AB/BA overexpression indicated that the highest band in p-Tyr and EphA4 blots (long arrow) appeared specific for EphA4, whereas the intermediate band (short arrow) corresponded to EphB4, the lower bands (arrowheads) a combination of both. Multiple bands may be due to differences in posttranslational modifications, in particular phosphorylation on multiple residues. Altogether, both chimeras appeared to be activated to similar levels by Fc fragments corresponding to cognate ligands. Note a slight activation by ephrinB2 Fc in controls, reflecting the abundance of endogenous receptors for ephrinB2 in the ectoderm.
(TIF)

Figure S4 **Eph kinase activity is required for tissue separation.** (A) KD variants of EphA4 (EphA4KD) and EphB4 (EphB4 KD) act as dominant negatives. EphA4KD expression in the mesoderm inhibited tissue separation and failed to rescue EphA4 depletion. Identical results were obtained by expression of EphB4KD in the ectoderm. (B) Ectopic induction of tissue separation between ectoderm explants by ephrinB2 Fc treatment was blocked by expression of KD EphB4. (B′) Induction of separation between mesoderm explants by ephrinB3 Fc treatment was similarly inhibited by expression of EphA4KD. ** indicates *p*<0.01 (Student's *t* test) compared to second columns. (C) Inhibition of separation by ephrinB2 depletion in the mesoderm can be rescued by treatment of the ectoderm with soluble ephrinB2 fragments (see Figure 1D). Expression of EphB4KD, however, blocked the ability of ectoderm cells to respond to ephrinB2. Similarly, soluble ephrinB3 Fc could not rescue separation between ephrinB3-depleted ectoderm and EphA4KD-expressing mesoderm. ** indicates *p*<0.01 (Student's *t* test) compared to the first columns. “ns,” not significant. (D) Inhibition of Eph phosphorylation. Left panel, EphB4. Control and EphB4KD-expressing ectoderm explants were treated with soluble ephrinB2 Fc fragments. Extracts were prepared and analyzed by immunoblot for p-EphB, total EphB4, and tubulin. Stimulation of EphB phosphorylation by ephinB2 Fc fragments was strongly inhibited by expression of EphB4KD. Right panel, EphA4. Significant phosphorylation of EphA4 was observed in untreated mesoderm explants, consistent with activation by one of its endogenous ligands, ephrinB2 (Figure 2), which is abundantly expressed in the mesoderm (Figure S1). Expression of EphA4KD in the mesoderm strongly decreased the p-EphA signal and failed to rescue p-EphA levels in EphA4MO-coinjected explants. Arrowhead, nonspecific band.
(TIF)

Figure S5 **Effect of cadherin levels and ephrin-Eph signaling on separation and tissue cohesion.** (A) Inhibition of separation upon cadherin overexpression and myosin inhibition. Tissue separation was inhibited by cadherin overexpression in the mesoderm but was rescued by increasing Eph signaling by treatment with soluble ephrinB2 Fc fragments. Separation was also strongly inhibited by treatment of wild-type explants with the myosin inhibitor blebbistatin. (B) Cadherin levels are not affected by Eph depletion. Immunofluorescence for C-cadherin of cryosections from whole embryos injected with control or anti-Eph morpholinos. GFP (immunostained in red) was used as the tracer. Note the strong disruption of the ectoderm–mesoderm boundary. (C–E) Tissue cohesion is decreased upon cadherin depletion or ectopic ephrin/Eph expression. Dissociated ectoderm and mesoderm cells were left to reaggregate under mild rotation for 1 h. (C) Effect of cadherin and/or EphB4 depletion on mesoderm reaggregation. (D) Effect of ephrin/Eph ectopic expression on mesoderm or ectoderm reaggregation. Ectoderm-specific ephrinB3 and EphB4 were expressed in the mesoderm, and mesoderm-specific ephrinB2 and EphA4 in the ectoderm. (E) Quantification of reaggregation. Two criteria were used, which gave similar results: the average aggregate area, which reflects the extent of aggregation, and area/perimeter ratio, which integrates both the size of the aggregates and their degree of compaction. Results from individual experiments were normalized using wild-type ectoderm/mesoderm as the reference (1.0) to account for batch-to-batch variation.
(TIF)

Figure S6 **Dose response of Eph activation by soluble ephrins.** Ectoderm explants were incubated for 30 min in the presence of different concentrations of ephrin-Fc fragments. Total extracts were analyzed by Western blot for p-Tyrosin levels. Phosphorylated Ephs represent a prominent band around 110 kDa (arrow) (see also Figure 2B). Samples were standardized for protein amount using β-catenin levels (as plasma membrane marker, also compared to total protein on Ponceau Red staining, not shown). (A) Example. (B) Average data from three experiments after subtraction of the endogenous signal, calculated from control condition (Fc). Curve fitting (one phase association) using GraphPad gave similar approximate Kds of ∼0.5–5 nM for both ephrins. Note that the curve for ephrinB2 was peculiar. Although its shape was compatible with calculation of the curve, it did not plateau, a feature that was reported in other cases and is not yet explained. The apparent Kd for ephrinB2 should be considered as a “global” affinity for all its ectodermal receptors (mostly EphBs). The apparent Kd for ephrinB3 can be considered to correspond to its Kd for EphA4, as it does not interact with EphBs (Figure 2D).
(TIF)

Figure S7 **Simulation of ephrin/Eph signaling in dorsal ectoderm and mesoderm and at the boundary.** (A) Principle of the simulation: the total signal output due to all the interactions between ephrins and Eph receptors at the tissue interface is computed (red double arrow), also taking into account the involvement of these molecules at homotypic contacts with surrounding cells in each tissue (pale double arrows). (A′) Diagrams of all the high affinity interactions between ephrins and Eph receptors at different cell contacts. Relative concentrations are symbolized by the size of the boxes, whereas the thickness of the red lines represents the relative intensities of the individual signals. (B and C) Apparent affinities and concentrations used for the simulation (basal values). (D) Effect of varying the range of concentrations and affinities on output for the stage 10.5 dorsal boundary. Each range of concentration was obtained by multiplying the values of table C by the indicated value. Selected affinities were varied as indicated. All other values were as in table B. The condition marked by a star corresponds to the basal values of tables B and C. (E) Results from Figure 4A, included for comparison. (F–J) Functional effect of manipulating ephrin and Eph levels: comparison of results from the separation assay (taken from Figures 2A, 3, S2A, and S2B) and of the corresponding simulation, using basal parameter values. The simulated boundary outputs are expressed as 100% of intensity signal at control ectoderm–mesoderm contacts.
(TIF)

Figure S8 **Effect of ephrin/Eph gain- and loss-of-function on ventral ectoderm–mesoderm separation.** (A) Summary of ephrin/Eph expression in stage 11 ventral tissues. The major differences compared to the dorsal side (Figure 1A) were the mesoderm enrichment of ephrinB1 and the even distribution of EphB4. (B) Inhibition of separation. Separation was assayed as in Figure 1B, but using ventral ectoderm and mesoderm (ventral lip) explants, dissected from stage 11 embryos. Separation was significantly impaired upon depletion of ephrinB3 and EphB2 on the ectoderm side, and for their corresponding partners EphA4 and ephrinB1 on the mesoderm side of the boundary. Note the stronger effect of ephrinB1 depletion compared to the results on the dorsal side (Figure S2A), consistent with its shift from an equal to an asymmetric distribution. ** indicate *p*<0.01 (Student's *t* test) compared to corresponding controls (white columns). (C) Induction of separation. Control ventral ectoderm explants normally mix. Significant separation was observed upon explant treatment with soluble Fc fragments corresponding to mesoderm-enriched ephrinB1 and B2, but not ephrinB3. EphrinB1-Fc–induced separation was significantly inhibited by EphB2 depletion, but not EphB4 depletion. The result is consistent with EphB2 acting as the preferred receptor for ephrinB1 (Figure 2B).
(TIF)

Movie S1 **Live imaging of contacts between single ectoderm and mesoderm cells.** A mixture of dissociated wild-type ectoderm cells expressing membrane-targeted GFP (green) and mesoderm cells expressing membrane-targeted Cherry (red) was plated on fibronectin. Newly re-established homotypic contacts between cells of the same tissue remained stable. Contacts between ectoderm and mesoderm cells underwent cycles of attachment (arrowheads) and repulsion/detachment (arrows). This movie shows maximum projection of 3 z planes of 0.2 µm distance.
(MP4)

Movie S2 **Live imaging of contact between single ectoderm and mesoderm cells treated with blebbistatin.** Mesoderm cells (red) and ectoderm cells (green) treated with 100 µM blebbistatin formed stable heterotypic contacts.
(MP4)

Movie S3 **Live imaging of contacts between single ectoderm and mesoderm cells overexpressing cadherin.** Mesoderm cells overexpressing cadherin (red) and ectoderm cells (green) maintained stable heterotypic contacts.
(MP4)

Movie S4 **Live imaging of contacts between control mesoderm cells.** Examples of formation of stable contacts between wild-type mesoderm cells (arrowheads).
(MP4)

Movie S5 **Live imaging of contacts between cadherin-depleted mesoderm cells.** Mesoderm cells injected with cadherin MO failed to establish stable contacts. The cell on the bottom right attaches (arrowheads) and detaches (arrows) several times from the neighboring cells. Arrowheads point to attachments, arrows to detachments.
(MP4)

Movie S6 **Live imaging of contacts between cadherin/EphB4-depleted mesoderm cells.** Mesoderm cells were prepared from embryos coinjected with cadherin MO and EphB4MO. Cells formed contact (arrowheads) and failed to retract. This demonstrates that the repulsion observed between cadherin MO cells was due to ephrin–Eph signaling.
(MP4)

Movie S7 **Live imaging of contacts between mesoderm cells overexpressing ephrinB3 and EphB4.** Ectopic coexpression of “ectodermal” ephrinB3 and EphB4 induced cycles of attachment and detachments between mesoderm cells. Arrowheads mark sites of contact and arrows subsequent retractions between the cell on the right side of the field and the two neighboring cells in the middle.
(MP4)

Text S1 **Supplementary materials and methods, and description of the simulation model of ephrin/Eph signaling.**
(DOCX)

## Abbreviations

KD: kinase dead
MO: morpholino oligonucleotide
p-MLC: phosphorylated myosin light chain
