# Supplementary figures and images for: Variable Combinations of Specific Ephrin Ligand/Eph Receptor Pairs Control Embryonic Tissue Separation
Source: PLoS Biol. 2014 Sep 23;12(9):e1001955. doi: 10.1371/journal.pbio.1001955 (PMC4172438; doi:10.1371/journal.pbio.1001955)

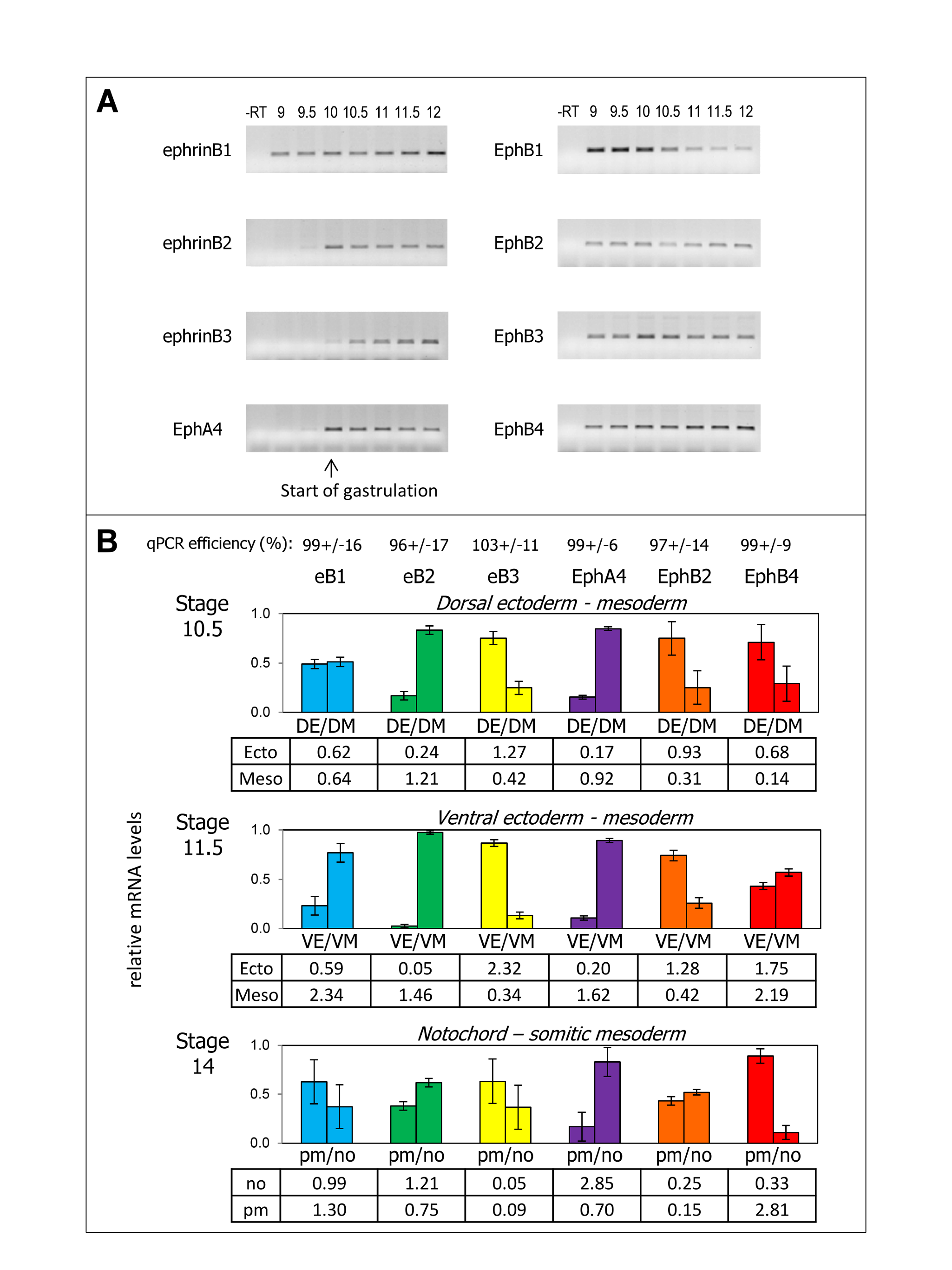

Supplement: Figure S1 — EphrinB1-3, EphA4, and EphB1–4 temporal expression during early Xenopus development and their relative tissue distribution. (A) General profile of total ephrin/Eph expression. RT-PCR was performed using mRNA extracted from whole embryos of the indicated stages. EphrinB1 and EphB1–4 are maternally expressed. EphrinB2, ephrinB3, and EphA4 are exclusively zygotic, starting at the onset of gastrulation (arrow). (B) Real-time quantitative RT-PCR of dissected tissues from stage 10.5 dorsal ectoderm and mesoderm, stage 11.5 ventral ectoderm and mesoderm, and stage 14 notochord and presomitic mesoderm. Bars express distribution between the two tissues. Error bars correspond to standard deviations (two independent series of samples). Numbers below each graph correspond to relative mRNA levels (arbitrary units), directly comparing all ephrins and Eph receptors for various tissues and stages. All values were corrected based on PCR efficiency. Average efficiencies are given above as %, with standard deviation. (TIF) [file pbio.1001955.s001.tif]

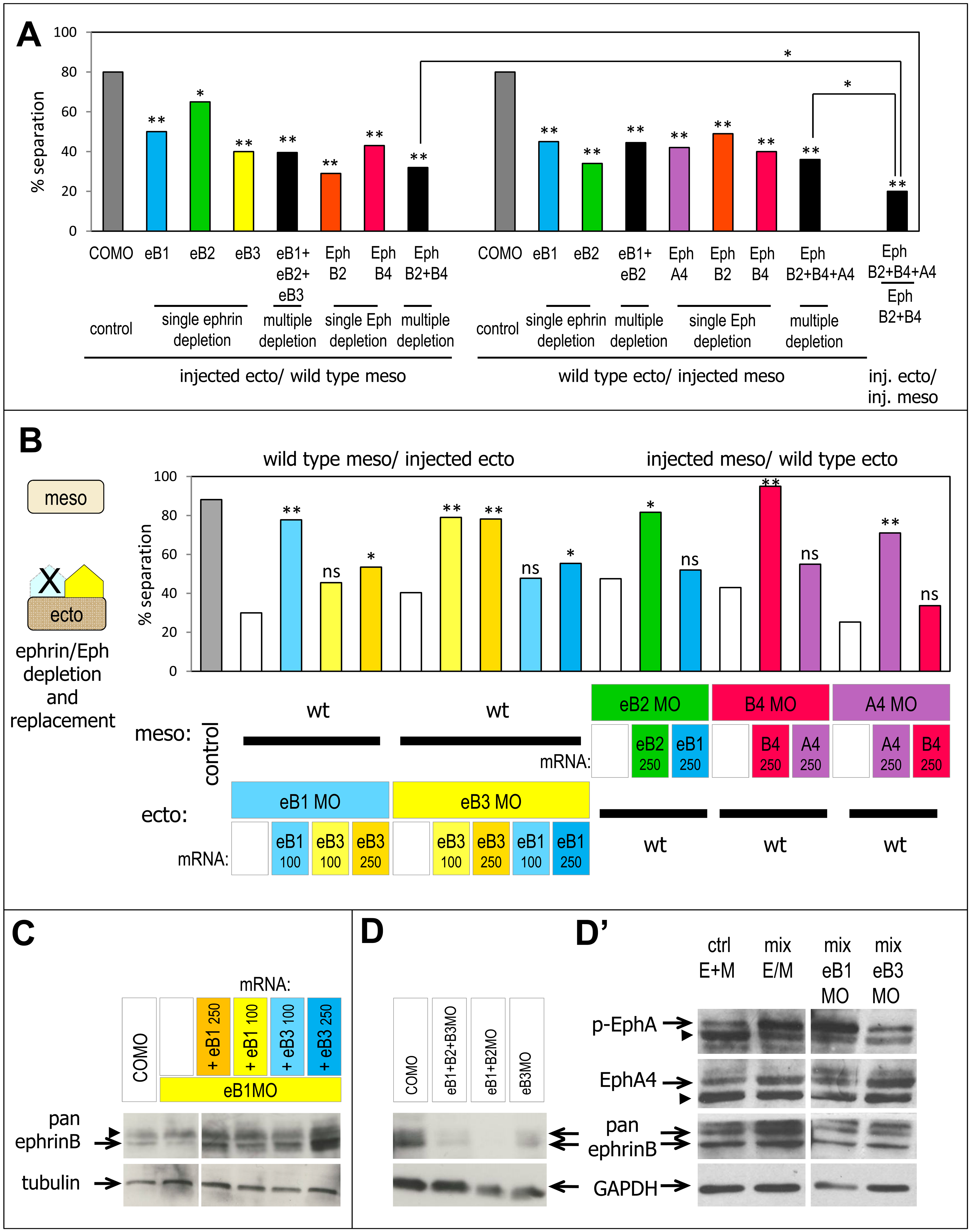

Supplement: Figure S2 — Multiple Ephrin/Eph play an additive role in tissue separation across the boundary. (A) Individual and multiple knockdowns. Single MO injections for each ephrin or Eph yielded a mixing phenotype, the penetrance of which related to the relative enrichment in each tissue (compare to Figure S1B). For instance, separation was strongly inhibited by ephrinB3 but not ephrinB2 depletion in the ectoderm, whereas ephrinB2 depletion had a strong effect in the mesoderm. Depletion of ephrinB1 gave intermediate inhibition in both tissues. The separation remaining after multiple ephrin or Eph depletions in one tissue was ∼30%–40%. Maximal inhibition could be reached in some cases by depletion of single molecules (e.g., ephrinB3 or EphB2 in the ectoderm). Depletion of Ephs on both sides led to almost complete inhibition of separation. * and ** indicate p<0.05 and p<0.01 (Student's t test) compared to controls (grey columns). (B) Each ephrin/Eph is specifically required and not replaceable. Individual ephrins and Eph receptors were depleted in the ectoderm or in the mesoderm, which induced inhibition of separation (white columns). Separation could be fully rescued by coinjection of mRNA (amounts indicated as pg/injection) coding for the corresponding ephrin/Eph (same colors). Only partial rescue was observed after heterotypical expression of other forms, even when expressed at high levels. * and ** indicate, respectively, p<0.05 and p<0.01 (Student's t test) compared to corresponding controls (white columns). “ns,” not significant. (C and D) Comparison by Western blot of ephrin levels in wild-type and manipulated ectoderm. (C) Conditions corresponding to the experiment presented in (B). Arrow points at specific ephrin band, decreasing in eB1MO. Both bands increased in ephrinB1/3 mRNA-injected embryos. Tubulin was used as the loading control. This blot is representative of three independent experiments. (D) Single and multiple ephrin depletion. Conditions are as in Figures 1D and S2A [file pbio.1001955.s002.tif]

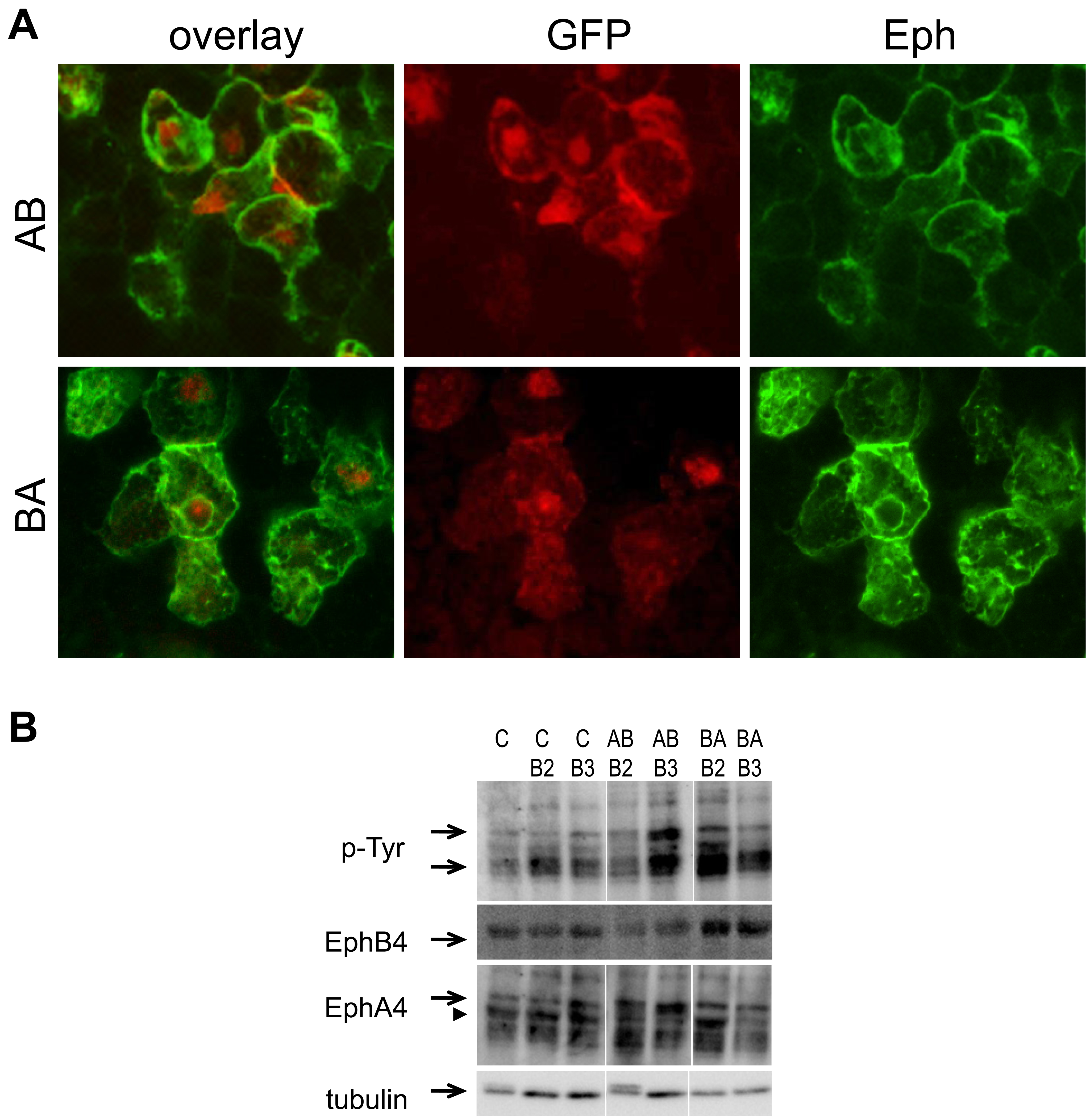

Supplement: Figure S3 — Expression of EphA4/B4 chimera constructs. (A) Immunofluorescence. Sections from ectoderm tissues expressing the AB or BA chimeras (see Figure 1 and main text) were immunolabeled using antibodies raised against the extracellular domains of EphA4 and EphB4, respectively. GFP, immunolabeled here in red, was coexpressed as a tracer. Both chimeras were well expressed at the plasma membrane. (B) Eph phosphorylation. Eph receptors appeared as major tyrosine-phosphorylated proteins in gastrula extracts, which allowed estimation of activation levels by blotting whole extract with an anti–p-Tyr antibody. Wild-type ectoderm explants or explants expressing AB or BA chimeras were incubated with ephrinB2 or ephrinB3 fragments for 30 min before extraction. Total extracts were analyzed by immunoblot using antibodies against p-Tyr and EphA4/B4 extracellular domains. EphB4 recognized a single band, but P-Tyr and EphA4 showed multiple bands. In the case of the anti-EphA4 antibody, this reflected cross-reactivity with other Eph receptors. However, comparison of controls and AB/BA overexpression indicated that the highest band in p-Tyr and EphA4 blots (long arrow) appeared specific for EphA4, whereas the intermediate band (short arrow) corresponded to EphB4, the lower bands (arrowheads) a combination of both. Multiple bands may be due to differences in posttranslational modifications, in particular phosphorylation on multiple residues. Altogether, both chimeras appeared to be activated to similar levels by Fc fragments corresponding to cognate ligands. Note a slight activation by ephrinB2 Fc in controls, reflecting the abundance of endogenous receptors for ephrinB2 in the ectoderm. (TIF) [file pbio.1001955.s003.tif]

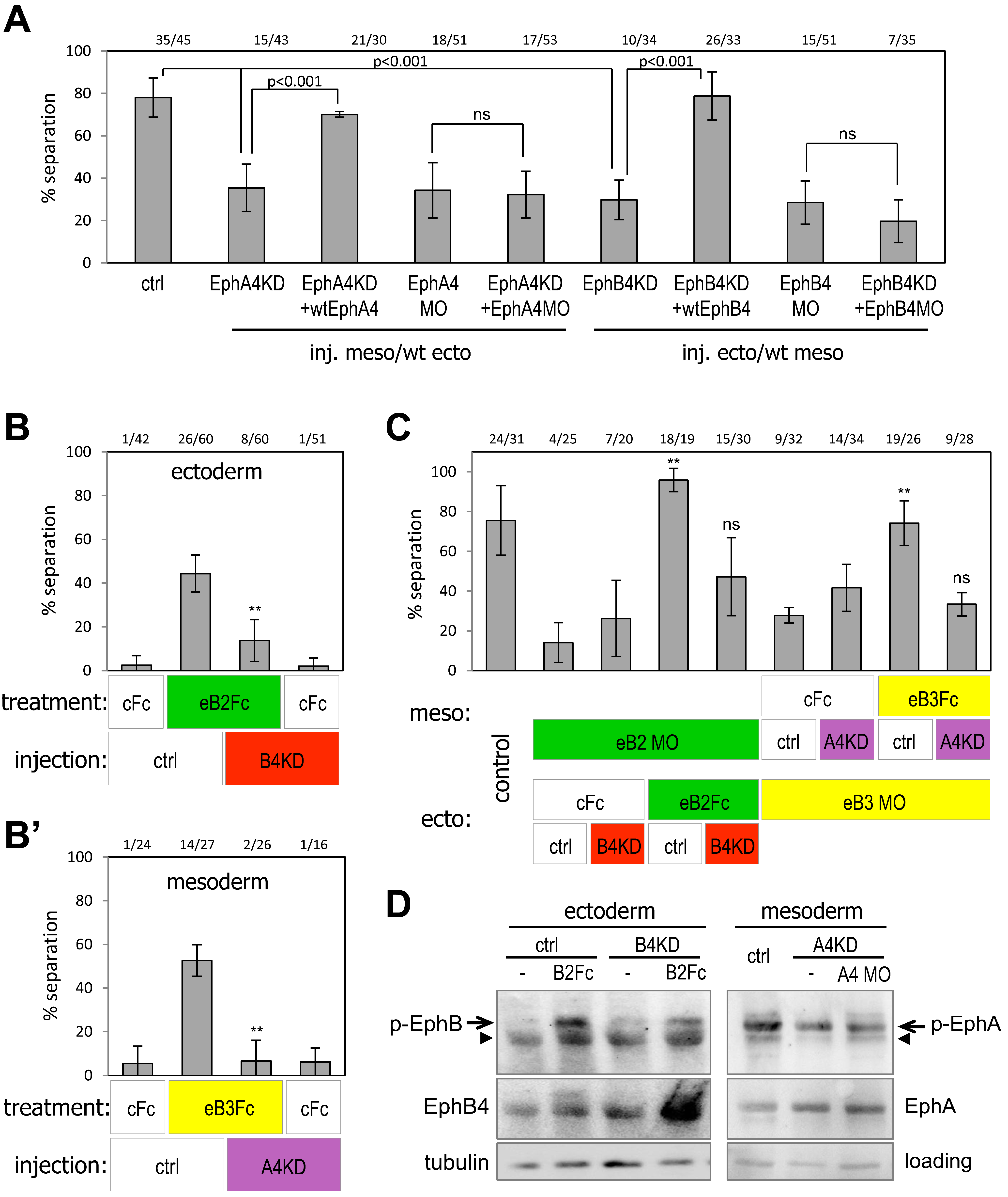

Supplement: Figure S4 — Eph kinase activity is required for tissue separation. (A) KD variants of EphA4 (EphA4KD) and EphB4 (EphB4 KD) act as dominant negatives. EphA4KD expression in the mesoderm inhibited tissue separation and failed to rescue EphA4 depletion. Identical results were obtained by expression of EphB4KD in the ectoderm. (B) Ectopic induction of tissue separation between ectoderm explants by ephrinB2 Fc treatment was blocked by expression of KD EphB4. (B′) Induction of separation between mesoderm explants by ephrinB3 Fc treatment was similarly inhibited by expression of EphA4KD. ** indicates p<0.01 (Student's t test) compared to second columns. (C) Inhibition of separation by ephrinB2 depletion in the mesoderm can be rescued by treatment of the ectoderm with soluble ephrinB2 fragments (see Figure 1D). Expression of EphB4KD, however, blocked the ability of ectoderm cells to respond to ephrinB2. Similarly, soluble ephrinB3 Fc could not rescue separation between ephrinB3-depleted ectoderm and EphA4KD-expressing mesoderm. ** indicates p<0.01 (Student's t test) compared to the first columns. “ns,” not significant. (D) Inhibition of Eph phosphorylation. Left panel, EphB4. Control and EphB4KD-expressing ectoderm explants were treated with soluble ephrinB2 Fc fragments. Extracts were prepared and analyzed by immunoblot for p-EphB, total EphB4, and tubulin. Stimulation of EphB phosphorylation by ephinB2 Fc fragments was strongly inhibited by expression of EphB4KD. Right panel, EphA4. Significant phosphorylation of EphA4 was observed in untreated mesoderm explants, consistent with activation by one of its endogenous ligands, ephrinB2 (Figure 2), which is abundantly expressed in the mesoderm (Figure S1). Expression of EphA4KD in the mesoderm strongly decreased the p-EphA signal and failed to rescue p-EphA levels in EphA4MO-coinjected explants. Arrowhead, nonspecific band. (TIF) [file pbio.1001955.s004.tif]

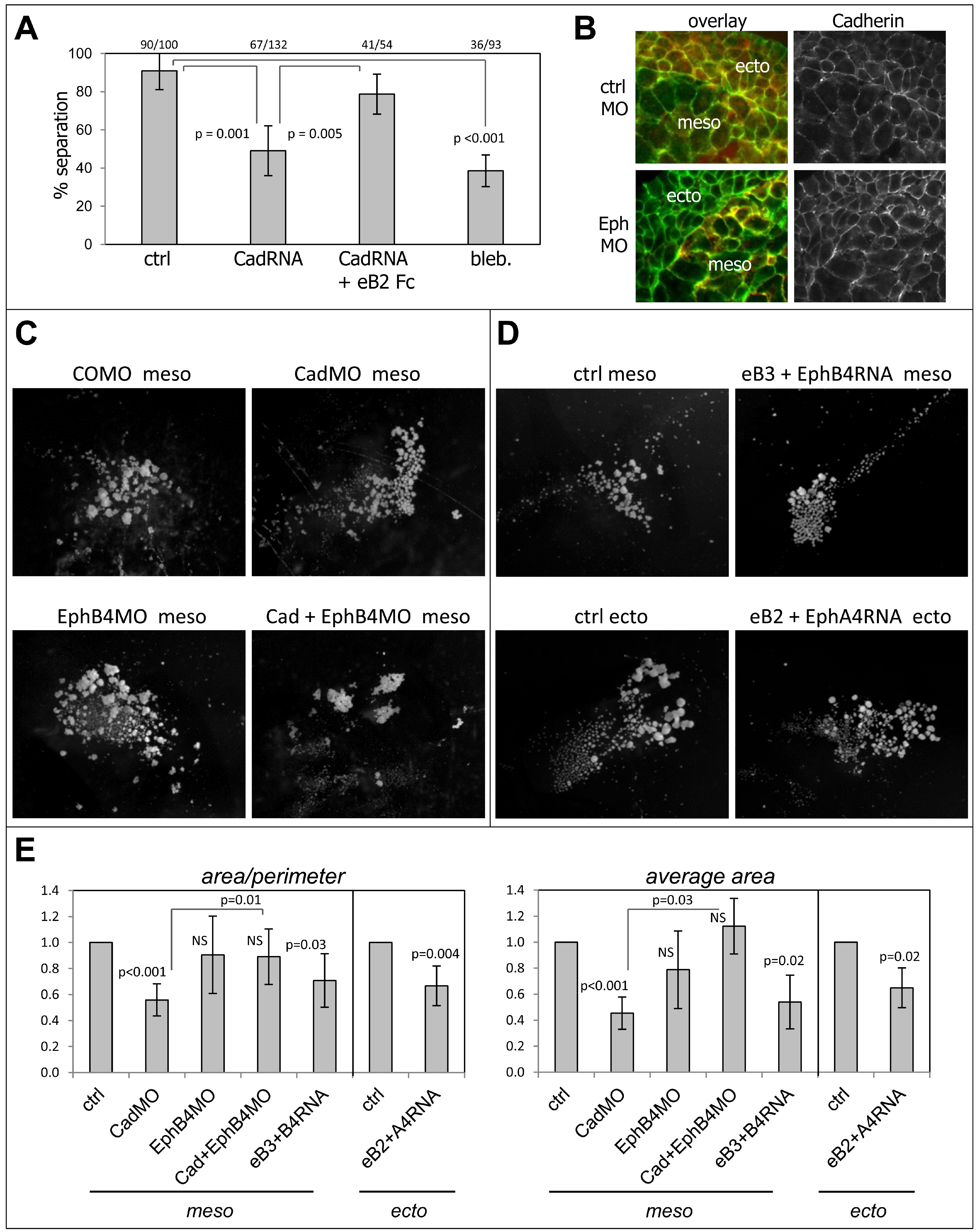

Supplement: Figure S5 — Effect of cadherin levels and ephrin-Eph signaling on separation and tissue cohesion. (A) Inhibition of separation upon cadherin overexpression and myosin inhibition. Tissue separation was inhibited by cadherin overexpression in the mesoderm but was rescued by increasing Eph signaling by treatment with soluble ephrinB2 Fc fragments. Separation was also strongly inhibited by treatment of wild-type explants with the myosin inhibitor blebbistatin. (B) Cadherin levels are not affected by Eph depletion. Immunofluorescence for C-cadherin of cryosections from whole embryos injected with control or anti-Eph morpholinos. GFP (immunostained in red) was used as the tracer. Note the strong disruption of the ectoderm–mesoderm boundary. (C–E) Tissue cohesion is decreased upon cadherin depletion or ectopic ephrin/Eph expression. Dissociated ectoderm and mesoderm cells were left to reaggregate under mild rotation for 1 h. (C) Effect of cadherin and/or EphB4 depletion on mesoderm reaggregation. (D) Effect of ephrin/Eph ectopic expression on mesoderm or ectoderm reaggregation. Ectoderm-specific ephrinB3 and EphB4 were expressed in the mesoderm, and mesoderm-specific ephrinB2 and EphA4 in the ectoderm. (E) Quantification of reaggregation. Two criteria were used, which gave similar results: the average aggregate area, which reflects the extent of aggregation, and area/perimeter ratio, which integrates both the size of the aggregates and their degree of compaction. Results from individual experiments were normalized using wild-type ectoderm/mesoderm as the reference (1.0) to account for batch-to-batch variation. (TIF) [file pbio.1001955.s005.tif]

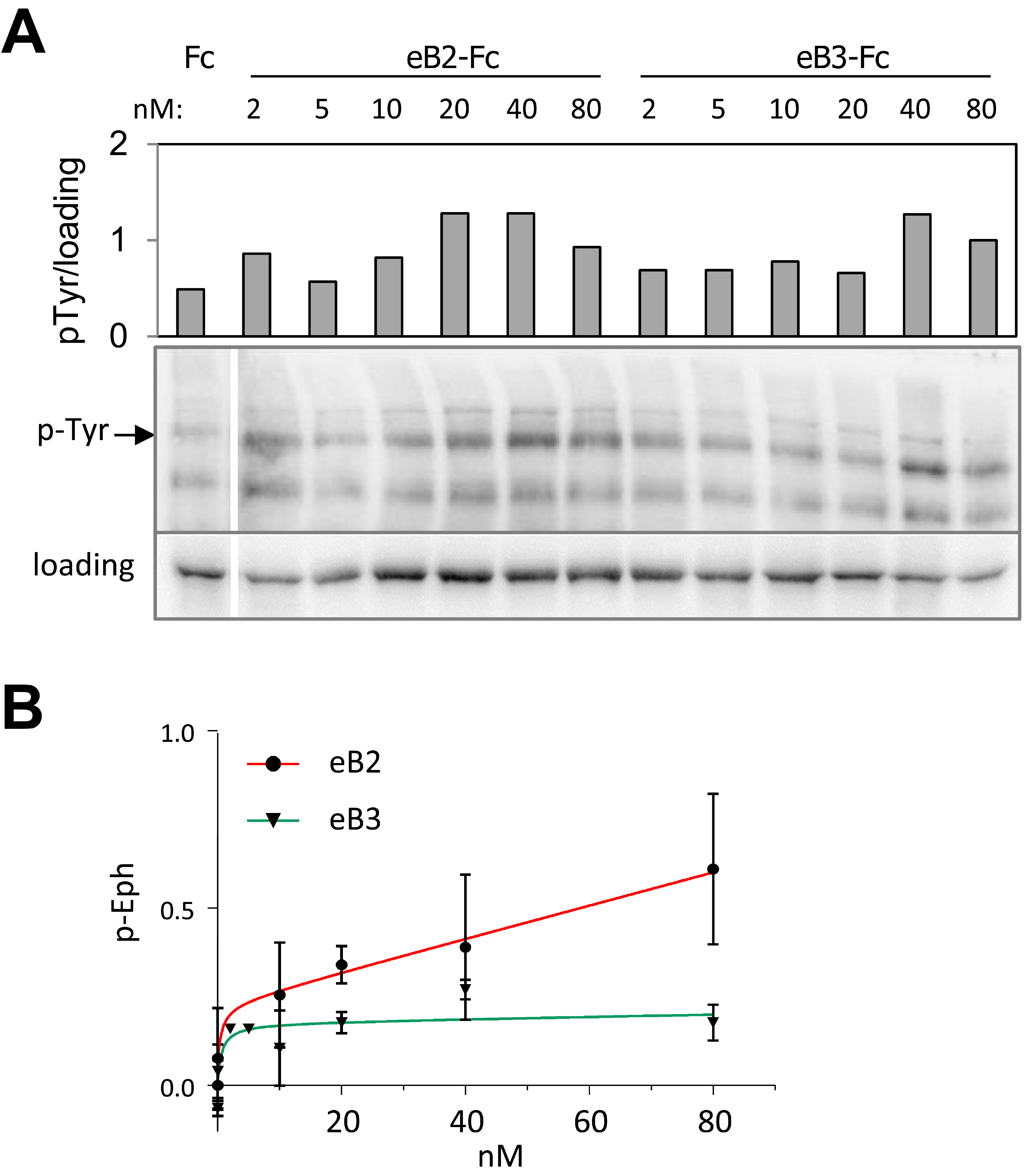

Supplement: Figure S6 — Dose response of Eph activation by soluble ephrins. Ectoderm explants were incubated for 30 min in the presence of different concentrations of ephrin-Fc fragments. Total extracts were analyzed by Western blot for p-Tyrosin levels. Phosphorylated Ephs represent a prominent band around 110 kDa (arrow) (see also Figure 2B). Samples were standardized for protein amount using β-catenin levels (as plasma membrane marker, also compared to total protein on Ponceau Red staining, not shown). (A) Example. (B) Average data from three experiments after subtraction of the endogenous signal, calculated from control condition (Fc). Curve fitting (one phase association) using GraphPad gave similar approximate Kds of ∼0.5–5 nM for both ephrins. Note that the curve for ephrinB2 was peculiar. Although its shape was compatible with calculation of the curve, it did not plateau, a feature that was reported in other cases and is not yet explained. The apparent Kd for ephrinB2 should be considered as a “global” affinity for all its ectodermal receptors (mostly EphBs). The apparent Kd for ephrinB3 can be considered to correspond to its Kd for EphA4, as it does not interact with EphBs (Figure 2D). (TIF) [file pbio.1001955.s006.tif]

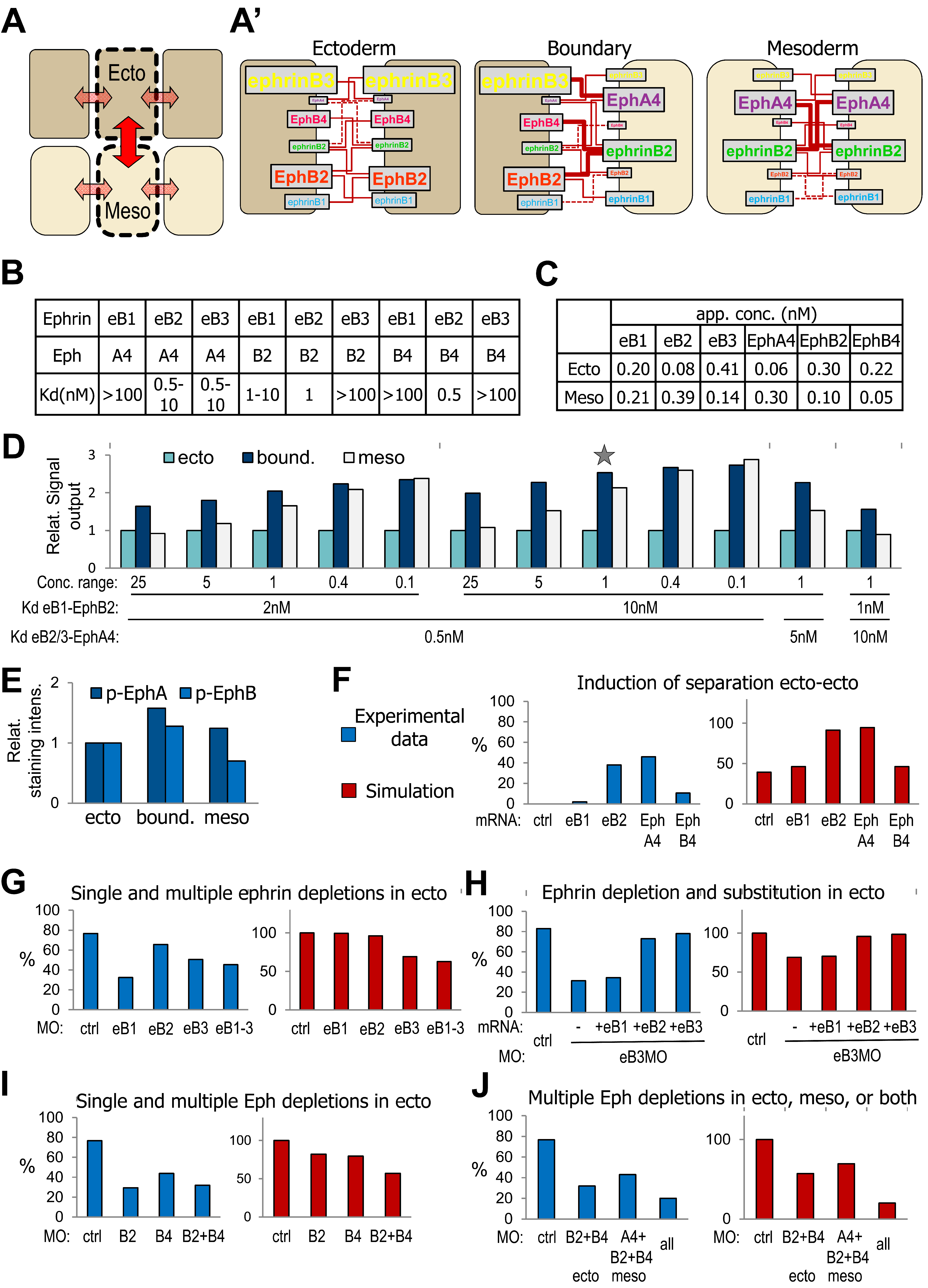

Supplement: Figure S7 — Simulation of ephrin/Eph signaling in dorsal ectoderm and mesoderm and at the boundary. (A) Principle of the simulation: the total signal output due to all the interactions between ephrins and Eph receptors at the tissue interface is computed (red double arrow), also taking into account the involvement of these molecules at homotypic contacts with surrounding cells in each tissue (pale double arrows). (A′) Diagrams of all the high affinity interactions between ephrins and Eph receptors at different cell contacts. Relative concentrations are symbolized by the size of the boxes, whereas the thickness of the red lines represents the relative intensities of the individual signals. (B and C) Apparent affinities and concentrations used for the simulation (basal values). (D) Effect of varying the range of concentrations and affinities on output for the stage 10.5 dorsal boundary. Each range of concentration was obtained by multiplying the values of table C by the indicated value. Selected affinities were varied as indicated. All other values were as in table B. The condition marked by a star corresponds to the basal values of tables B and C. (E) Results from Figure 4A, included for comparison. (F–J) Functional effect of manipulating ephrin and Eph levels: comparison of results from the separation assay (taken from Figures 2A, 3, S2A, and S2B) and of the corresponding simulation, using basal parameter values. The simulated boundary outputs are expressed as 100% of intensity signal at control ectoderm–mesoderm contacts. (TIF) [file pbio.1001955.s007.tif]

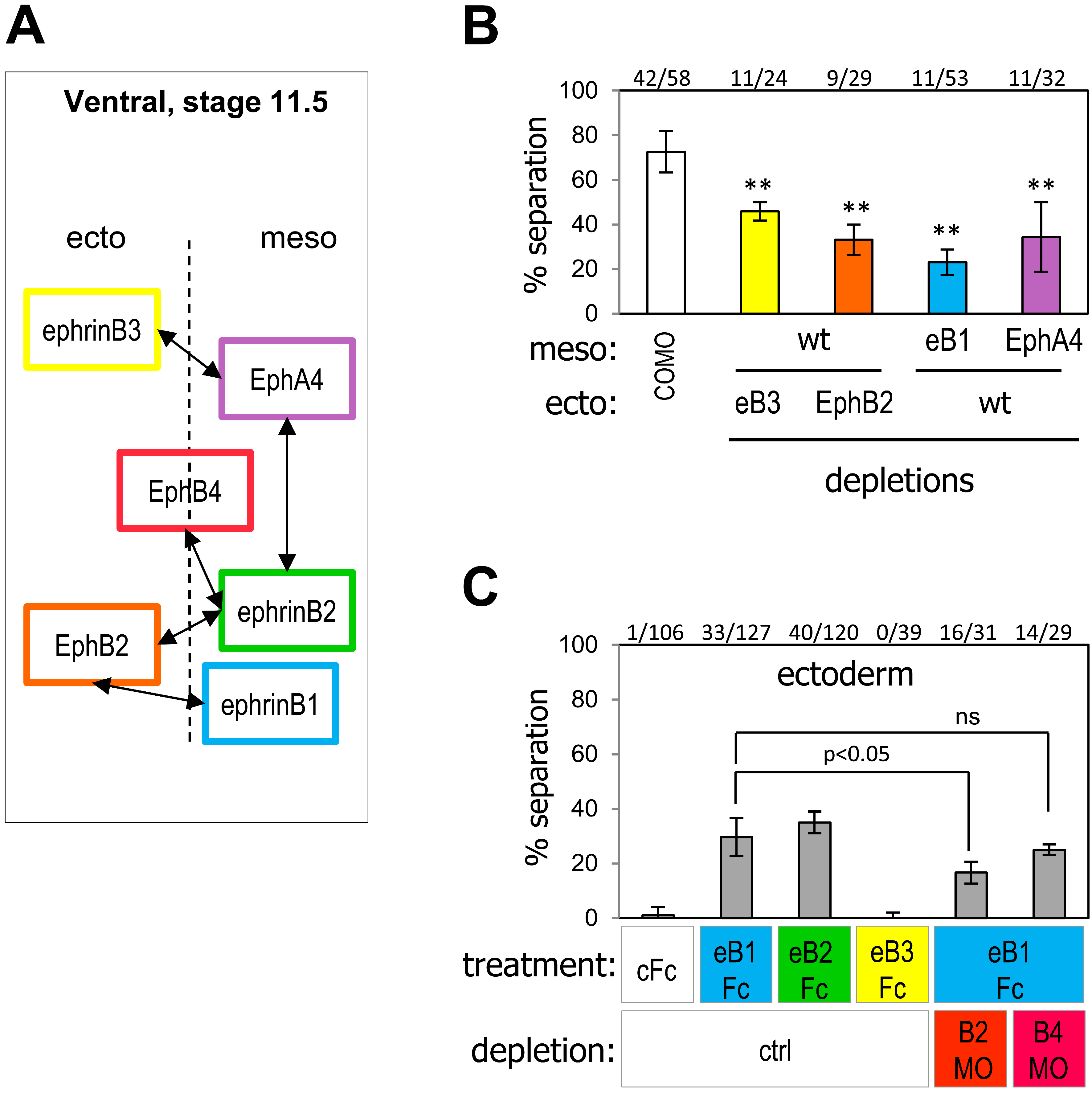

Supplement: Figure S8 — Effect of ephrin/Eph gain- and loss-of-function on ventral ectoderm–mesoderm separation. (A) Summary of ephrin/Eph expression in stage 11 ventral tissues. The major differences compared to the dorsal side (Figure 1A) were the mesoderm enrichment of ephrinB1 and the even distribution of EphB4. (B) Inhibition of separation. Separation was assayed as in Figure 1B, but using ventral ectoderm and mesoderm (ventral lip) explants, dissected from stage 11 embryos. Separation was significantly impaired upon depletion of ephrinB3 and EphB2 on the ectoderm side, and for their corresponding partners EphA4 and ephrinB1 on the mesoderm side of the boundary. Note the stronger effect of ephrinB1 depletion compared to the results on the dorsal side (Figure S2A), consistent with its shift from an equal to an asymmetric distribution. ** indicate p<0.01 (Student's t test) compared to corresponding controls (white columns). (C) Induction of separation. Control ventral ectoderm explants normally mix. Significant separation was observed upon explant treatment with soluble Fc fragments corresponding to mesoderm-enriched ephrinB1 and B2, but not ephrinB3. EphrinB1-Fc–induced separation was significantly inhibited by EphB2 depletion, but not EphB4 depletion. The result is consistent with EphB2 acting as the preferred receptor for ephrinB1 (Figure 2B). (TIF) [file pbio.1001955.s008.tif]
