# Supplementary material for: Variable Combinations of Specific Ephrin Ligand/Eph Receptor Pairs Control Embryonic Tissue Separation
Source: PLoS Biol. 2014 Sep 23;12(9):e1001955. doi: 10.1371/journal.pbio.1001955 (PMC4172438; doi:10.1371/journal.pbio.1001955)
Supplement: Text S1 — Supplementary materials and methods, and description of the simulation model of ephrin/Eph signaling. (DOCX) [file pbio.1001955.s016.docx]

**Text S1**

**Supplementary materials and methods**

All animal studies were approved by the McGill University Animal Care Committee, permit #4869 “Cellular mechanisms of embryonic boundary formation” and the University of Toronto Animal Care Committee, permit # 20010074 “Analysis of gastrulation movements in Xenopus”.

**Morpholino anti-sense oligonucleotide sequences**

| Target | Sequence | |  |
| --- | --- | --- | --- |
| ephrinB1 |  | GGAGCCCTTCCATCCGCACAGGTGG |  |
| ephrinB2 |  | ACACCGAGTCCCCGCTCAGTGCCAT |  |
| ephrinB2a |  | ACACCGAGTCCCCGCTCAGTGCCAT |  |
| ephrinB2b |  | ACACCGAGTCCCCGCTCAGTGCCAT |  |
| ephrinB3 |  | CGGGAAAACATGCTGATTAAAGGGC |  |
| EphA4 |  | AGATGCCATGTACAATCCCAGCCAT |  |
| EphB2 |  | CCCCATAGTCCTGGAAGGCCAGGTA |  |
| EphB4a |  | ACAGGAGGAGGAGCCAGAGATCCAT |  |
| control |  | CCTCTTACCTCAGTTACAATTTATA |  |

**Amounts of morpholinos injected**

| **Morpholinos** | **ng injected per blastomere** |
| --- | --- |
| Control | 40 |
| ephrinB1 | 40 |
| ephrinB2 | 40 |
| ephrinB2a | 40 |
| ephrinB2b | 40 |
| ephrinB3 | 40 |
| EphA4 | 40 |
| EphB2 | 40 |
| EphB4a | 30 |
| ephrinB1 + ephrinB2 + ephrinB3 | 20+20 +20 |
| ephrinB1+ephrinB2 | 20+20 |
| EphB2+ EphB4 | 20+20 |
| EphB2 +EphB4 + EphA4 | 20+20 +20 |

**List of mRNAs and amounts injected**

| **mRNAs** | | | |  |
| --- | --- | --- | --- | --- |
|  | Species | vector | pg injected per blastomere |  |
| EphrinB1 | Xenopus | pCS2+ | 100-250 |  |
| EphrinB2 | Xenopus | pCS2+ | 100-250 |  |
| EphrinB3 | Xenopus | pCS2+ | 100-250 |  |
| EphB4 | Xenopus | pCS2 | 400 |  |
| EphA4 | Xenopus | pBluscript KS | 500-1000 |  |
| EphB4* | Xenopus | pCS2 | 250-500 |  |
| EphA4* | Xenopus | pCS2 | 250-500 |  |
| EphA4B4 | Xenopus | pCS2 | 250-500 |  |
| EphB4A4 | Xenopus | pCS2 | 250-500 |  |
| EphB4KD | Xenopus | pCS2 | 800 |  |
| EphA4KD | Mouse | pCS2+ | 600 |  |
| EphA4-YFP | Chicken | pCS2 | 500-1000 |  |
| membrane GFP |  | pCS2+ | 100-250 |  |
| membrane Cherry |  | pCS2+ | 100-250 |  |
| Alk4* | Xenopus | pCS2+ | 1000 |  |
| β-catenin | Xenopus | pSP36T | 120 |  |

**Primers used for RT-PCR**

| Primer pair | Sequence |
| --- | --- |
| EphrinB1 | Forward: GCCCTAGCAAAGAGGCTGA  Reverse:CCGTGGGTTTATCAGACAGG |
| EphrinB2(a+b) | Forward: TGGCTCTTTAGAGGGTGTGG  Reverse:CGCCTCGATGGTTATGAAAG |
| EphrinB3 | Forward:CTGCTGCTGGTCTTTGGAGT  Reverse:CATGGCCGTTGTTGTTACCT |
| EphA4(a+b) | Forward: GGTGGCTTCTGTGCTTGATT  Reverse: GACTGCTTCCAAGCTGGTGT |
| EphB1 | Forward: AGCAGGATTATCGCCTTCCT  Reverse: GCCAGCAGTCCAGCATAAGT |
| EphB2 | Forward: GTAGCACCGCTGTCTTCAGG  Reverse: CCTTGTACTGCGCCATCTTT |
| EphB3 | Forward: TTCTGAGCGCTGGATTCACT  Reverse: GCAGGTCCTCAGCTGTCATT |
| EphB4(a+b) | Forward: GTGTCCGACTTTGGCCTCT  Reverse: GGATCTTCCCTCCCAGACAA |

**Simulation of ephrin/Eph signaling in dorsal ectoderm and mesoderm and at the boundary.**

***Principle and assumptions***

The system involves three ephrins (B1,B2,B3) and three Eph receptors (EphA4,B2,B4) expressed at different levels in ectoderm and mesoderm cells. Ephrins can interact with cognate receptors within the tissues (homotypic ectoderm-ectoderm and mesoderm-mesoderm contacts) and across the tissue interface (heterotypic ectoderm-mesoderm contacts) (Figure 7A, respectively pale and red double arrows). The system was considered as a whole, taking simultaneously into account all the reactions occurring at the three different types of contacts (Figure S7A’). The ephrin-Eph interactions are dictated by “apparent affinities” (Kd) according to the law of mass equation

[ephrin-Eph] = [ephrin_free_][Eph_free_]/K_d_. The equations write as following:

where, for example, the notation [X∙y ]_EM_ means that the complex X∙y is formed from the Ephrin receptor X in the ectoderm and the ephrin ligand y in the mesoderm.

This set of equations should be combined with the equations of the conservation of the total concentrations of Ephrin receptors and ephrin ligands in each tissue,:

The resulting signal outputs at the various contacts have been computed numerically by solving this system of 48 polynomial equations using Maple. These outputs are calculated as the sum of all receptor-ligand complexes formed at each type of contact :

Note that the system considered is assumed to be at steady-state, with ephrins and Ephs distributing between the different contacts according to partner availability. This assumption is motivated by the long incubation times of the functional assays (60min), while ephrin-Eph-mediated reactions are detected within a few minutes ([1] and unpublished data) such that 60 min should be largely sufficient for components to diffuse and equilibrate along the cell surface.

Ephrin-Eph signaling is thought to depend on clustering, which appears to be spontaneous for ephrins and ligand-induces for Eph receptors [2]. However, the size of the clusters and the effect on diffusion and affinities remains unclear. It is reasonable to assume that these effects were of similar magnitude for all pairs, and may not impact significantly on the relative contribution of each pair. Inhibition through lateral ephrin-Eph cis-interactions was also reported, but so far only for ephrinAs [3], and may only occur at very high expression levels. Opposite evidence for complete separation of ephrins and Ephs expressed on the same membrane was also reported [4]. In the absence of more definitive evidence, cis-interactions were not taken into account in this computation. Induction of separation by Fc-Eph fragments demonstrated the existence of reverse signaling (Figure 2A”). Indirect data suggest that ephrinB3 may transmit more efficient reverse signaling than other ephrins in these tissues. Yet in the absence of direct way to monitor this signal, we conservatively considered it to be similar for all pairs. Note that inputting a stronger ephrinB3 reverse signaling would be predicted to increase the difference between boundary and tissue outputs, due to the strong asymmetry of the ephrinB3-EphA4 pair.

***Parameter values***

A set of “basal values”, presented in Figure S7B and C, was established based on the following information and considerations. Note that “apparent” affinities and concentrations were used here as purely operational terms.

Apparent affinities

Kd values presented in Figure 7B were based on published values [5], with some adjustments prompted by the IP results (Figure 2B) and on the *in vivo* dose responses from Figure S6. These latter experiments gave an approximate global Kd for ephrinB2 toward its receptors (which in the ectoderm are mostly EphBs) of ~0.5-5nM, thus similar to published values (0.5-2nM). Information about ephrinB2-EphA4 and ephrinB3-EphA4 affinities was scarce and imprecise. The reported Kds are relatively high (respectively 9nM and >6nM?), which is inconsistent with the fact that they are among the most frequent functional pairs found in physiological contexts [6]. Both ephrins activated EphA4 very effectively in our experiments, and the dose response for ephrinB3 also supported a lower Kd (0.5-5nM, Figure S6). Published ephrinB1-EphB2 and ephrinB2-EphB2 Kds were respectively ~0.6nM and 0.8nM. The latter was compatible with our depletion experiments, but not with the biochemical data, where ephrinB1 activated EphB2 much less than ephrinB2 (Figure 2D). We consider reasonable to assume that steady-state levels of phosphorylated Eph receptors correctly reflect the degree of activation of the pathway, although differences in other parameters such as turnover or dephosphorylation may perhaps distort this relationship. We could not successfully fit the dose response for ephrinB1 to estimate an *in vivo* Kd (data not shown).

For these ambiguous cases, we tested both published values and adjusted values (range indicated in Figure S7B). They were all compatible with the system’s normal behavior (Figure S7D).

Ephrin and Eph apparent concentrations

Ephrin and Eph protein levels could not be directly estimated, due to lack of antibodies with sufficient isoform specificity. Relative concentrations of the various ephrins and Ephs were assumed to be proportional to mRNA levels determined by qPCR (Figure S1B). These values were converted into apparent protein concentrations by multiplication by an arbitrary factor (Figure S7C). The concentration range was set as follows: Global endogenous activity in the ectoderm was estimated based on the levels of phosphorylated Ephs in unstimulated ectoderm explants (control condition, Figure S6). The estimate used the dose responses to exogenous ephrins as reference, and took into account the fact that soluble ligands could only access the surface of the explants, while the endogenous signals where generated over the larger contact surface of inside the explants. We thus calculated that the “apparent concentration” of endogenous ephrins should be between one tenth of nanomalor and a few nanomolar. We set a lower “basal range of concentrations”, defined as “concentration range 1” (Figure S7C, D). Other ranges were then obtained by multiplication of all concentrations by a given factor (0.1 to 25 folds, Figure S7C, D).

Despite the fact that additional parameters (e.g. rate of translation and endocytosis) may differentially affect the relationship between mRNA levels and expression at the cell surface, we did not detect in our experiments any obvious deviation, which would suggest that contribution of a particular ephrin or Eph may be drastically disproportional to its mRNA level. In particular, single ephrin or Eph depletions (Figure S2) inhibited separation to a degree well in agreement with their predicted relative abundance (and available interacting partners on the opposite side of the boundary). As another example, the relative lower levels of EphA4 compared to EphBs in the ectoderm were compatible with the lower levels of global Eph phosphorylation for ephrinB3 stimulation compared to ephrinB2 (Figure S6).

***Results and discussion***

***General outputs and simulation of experimental manipulations***

The total signal output at the boundary was found to be higher than the signals within each of the two tissues, in agreement with the model built on our experimental data. The system appeared robust: the ephrin-Eph signal remained higher at the boundary over a range of concentrations that encompassed our most conservative estimates (Figure S7D). The system appeared also surprisingly resistant to variations in Kd values. Indeed, as shown in Figure S7D, the model remained valid for all simulations calculated using either published Kds or adjusted Kds based on our *in vivo* experiments, thus over a 10-20 fold range.

Figure S7F-J shows the comparison of the results of several experiments with the corresponding predictions from the simulation. They include overexpression of ephrins and Ephs in the ectoderm and test for ectopic separation of two ectoderm tissues (F), single and multiple depletions of ephrins in the ectoderm (G), depletion and substitution (H), single and multiple depletions of Eph receptors in the ectoderm, mesoderm, and both (I and J). In most cases, the patterns were strikingly similar, confirming that simulation reproduced extremely well most aspects of the system.

The broad range of concentrations and affinities compatible with a stronger repulsive signal at the boundary suggested that the model could accommodate not only the above-mentioned inconsistencies and methodological inaccuracies, but also deviations from a simple direct relationship between ligand-receptor binding and repulsion. Multiple factors are indeed expected to influence this relationship, including ligand and receptor clustering, receptor phosphorylation/ dephosphorylation, endocytosis and recycling, transmission of the signal into myosin activation. Yet our results generally confirmed a surprisingly good correlation between ligand/receptor levels, combined with theoretical affinity values, and experimental read-outs at various levels, i.e. Eph phosphorylation (Figure 4A,B), myosin phosphorylation (Figure 4C), cell repulsion (Figure 5) and overall tissue separation (Figures 1-3), implying that the number of ligand-receptor interactions that can be established at a cell contact is likely to be the major determinant of the system. Since binding affinities and levels are all relatively similar, the system can be approximated reasonably well by considering that all functional pairs may contribute to a similar extent. The key factor then remains the general distribution of the various partners, including a number of complementary asymmetric patterns.

Obviously one expects smooth gastrulation of the real embryo to rely on more precise tuning of the system, in particular to achieve the right balance between adhesion and repulsion required for mesoderm to migrate on the ectoderm surface during involution, and at the same time confer to the two tissues properties responsible for their specific morphogenetic movements. In this context, feeding the simulation with different parameter values gave different relative signals in the ectoderm and in the mesoderm (Figure S7D), an effect that can be explained by the balance between EphA4, dominant in the mesoderm, and EphBs, which control repulsion on the ectoderm side, Figures 2C,4A, and S7 A’,E). Available data tend to suggest that repulsion is probably stronger in the mesoderm. In particular, ephrin depletion significantly increased mesoderm but not ectoderm cohesion (Rohani et al, 2011), and the portion of pMLC signal intensity that appears to be ephrin/Eph-dependent is clearly higher in the mesoderm (compare control and Eph-depletion in Figure 4C). Yet the system is complex and additional experiments will be needed to solve this question. The simulation warns us that the behavior of system is not necessarily intuitive: the fact that experimental Eph depletions gave stronger inhibition in the ectoderm than in the mesoderm could be superficially interpreted as evidence for stronger Eph activation in the ectoderm. Yet, according to the simulation, these results are also compatible with the basal parameters (compare 2^nd^ and 3^rd^ columns in Figure S7J), i.e. conditions that predict a significantly higher total output in the mesoderm (Figure S7D).

Some conditions were not satisfactorily reproduced, providing useful information about the limits of this model. It failed in particular to mimic the relatively strong inhibition of separation caused by ephrinB1 depletion (Figure S7G), at least when inputting a Kd compatible with its low binding to EphB2 in our biochemical experiments (Figure 2D). One possible explanation could be the existence of yet another Eph receptor for ephrinB1. Another smaller inconsistency concerns the ephrinB2-EphA4 and ephrinB3-EphA4 pairs: we set the latter similar to the ephrinB2-EphA4 Kd, by lack of more precise data [5]. Under these conditions, ephrinB2 would be predicted to substitute for ephrinB3 in all its functions, and to be generally more influential, due to its ability to also interact with EphBs. EphrinB2 did indeed rescue very efficiently loss of ephrinB3 (Figures 1D and 3). Yet only ephrinB3 could induce separation between two mesoderm tissues, suggesting that ephrinB2 cannot mimic all aspects of ephrinB3 function (Figure 2A’). Finally, simulations of gain-of-function experiments (including rescues) gave generally higher levels of activation than experimentally observed (Figure 7F and data not shown).

It is probably not fortuitous that the major inconsistencies involved those ephrin-Eph pairs for which Kd values were particularly uncertain. Yet, adjusting Kds may not be sufficient to fully account for the experimental results, and other factors should also be taken into consideration: For instance, our experiments involved long stimulations, which aimed at mimicking the physiological situation at the boundary. This allowed us to make the reasonable assumption that the system was near steady-state. It is however quite possible that some receptors may have different turnover and that average levels of phosphorylated receptors may not always reflect the actual intensity of the signal. It is also likely that responses may be strongly non-linear, which could explain in particular why simulation of overexpression gave too high outputs.

**References**

1. Rohani N, Canty L, Luu O, Fagotto F, Winklbauer R (2011) EphrinB/EphB signaling controls embryonic germ layer separation by contact-induced cell detachment. PLoS Biol 9: e1000597.

2. Himanen JP, Yermekbayeva L, Janes PW, Walker JR, Xu K, et al. (2010) Architecture of Eph receptor clusters. Proc Natl Acad Sci USA 107: 10860-10865.

3. Carvalho RF, Beutler M, Marler KJM, Knoll B, Becker-Barroso E, et al. (2006) Silencing of EphA3 through a cis interaction with ephrinA5. Nat Neurosci 9: 322-330.

4. Kao T-J, Kania A (2011) Ephrin-Mediated cis-Attenuation of Eph Receptor Signaling Is Essential for Spinal Motor Axon Guidance. Neuron 71: 76-91.

5. Blits-Huizinga CT, Nelersa CM, Malhotra A, Liebl DJ (2004) ephrins and their receptors: binding versus biology. IUBMB Life 56: 257-265.

6. Pasquale EB (2004) Eph-ephrin promiscuity is now crystal clear. Nat Neurosci 7: 417-418.

**Legends supplementary Figures**

**Figure S1**

**EphrinB1-3, EphA4, and EphB1–4 temporal expression during early Xenopus development and their relative tissue distribution.** (A) General profile of total ephrin/Eph expression. RT-PCR was performed using mRNA extracted from whole embryos of the indicated stages. EphrinB1 and EphB1–4 are maternally expressed. EphrinB2, ephrinB3, and EphA4 are exclusively zygotic, starting at the onset of gastrulation (arrow). (B) Real-time quantitative RT-PCR of dissected tissues from stage 10.5 dorsal ectoderm and mesoderm, stage 11.5 ventral ectoderm and mesoderm, and stage 14 notochord and presomitic mesoderm. Bars express distribution between the two tissues. Error bars correspond to standard deviations (two independent series of samples). Numbers below each graph correspond to relative mRNA levels (arbitrary units), directly comparing all ephrins and Eph receptors for various tissues and stages. All values were corrected based on PCR efficiency. Average efficiencies are given above as %, with standard deviation.

**Figure S2**

**Multiple Ephrin/Eph play an additive role in tissue separation across the boundary.** (A) Individual and multiple knockdowns. Single MO injections for each ephrin or Eph yielded a mixing phenotype, the penetrance of which related to the relative enrichment in each tissue (compare to Figure S1B). For instance, separation was strongly inhibited by ephrinB3 but not ephrinB2 depletion in the ectoderm, whereas ephrinB2 depletion had a strong effect in the mesoderm. Depletion of ephrinB1 gave intermediate inhibition in both tissues. The separation remaining after multiple ephrin or Eph depletions in one tissue was ~30%–40%. Maximal inhibition could be reached in some cases by depletion of single molecules (e.g., ephrinB3 or EphB2 in the ectoderm). Depletion of Ephs on both sides led to almost complete inhibition of separation. * and ** indicate *p* < 0.05 and *p* < 0.01 (Student’s *t* test) compared to controls (grey columns). (B) Each ephrin/Eph is specifically required and not replaceable. Individual ephrins and Eph receptors were depleted in the ectoderm or in the mesoderm, which induced inhibition of separation (white columns). Separation could be fully rescued by coinjection of mRNA (amounts indicated as pg/injection) coding for the corresponding ephrin/Eph (same colors). Only partial rescue was observed after heterotypical expression of other forms, even when expressed at high levels. * and ** indicate, respectively, *p* < 0.05 and *p* < 0.01 (Student’s *t* test) compared to corresponding controls (white columns). “ns,” not significant. (C and D) Comparison by Western blot of ephrin levels in wild-type and manipulated ectoderm. (C) Conditions corresponding to the experiment presented in (B). Arrow points at specific ephrin band, decreasing in eB1MO. Both bands increased in ephrinB1/3 mRNA-injected embryos. Tubulin was used as the loading control. This blot is representative of three independent experiments. (D) Single and multiple ephrin depletion. Conditions are as in Figures 1D and S2A. In this blot, ephrinBs appear as multiple bands (arrows). (D’) Conditions corresponding to experiment presented in Figure 3B. p-EphA4 (arrow) was increased in mixed aggregates (mix E/M) compared to ectoderm + mesoderm aggregates (E+M). This increase was abolished by depletion of ephrinB3 (eB3MO) but not ephrinB1 (eB1MO). Arrowheads, nonspecific bands.

**Figure S3**

**Expression of EphA4/B4 chimera constructs.** (A) Immunofluorescence. Sections from ectoderm tissues expressing the AB or BA chimeras (see Figure 1 and main text) were immunolabelled using antibodies raised against the extracellular domains of EphA4 and EphB4, respectively. GFP, immunolabelled here in red, was coexpressed as a tracer. Both chimeras were well expressed at the plasma membrane. (B) Eph phosphorylation. Eph receptors appeared as major tyrosine-phosphorylated proteins in gastrula extracts, which allowed estimation of activation levels by blotting whole extract with an anti–p-Tyr antibody. Wild-type ectoderm explants or explants expressing AB or BA chimeras were incubated with ephrinB2 or ephrinB3 fragments for 30 min before extraction. Total extracts were analyzed by immunoblot using antibodies against p-Tyr and EphA4/B4 extracellular domains. EphB4 recognized a single band, but P-Tyr and EphA4 showed multiple bands. In the case of the anti-EphA4 antibody, this reflected cross-reactivity with other Eph receptors. However, comparison of controls and AB/BA overexpression indicated that the highest band in p-Tyr and EphA4 blots (long arrow) appeared specific for EphA4, whereas the intermediate band (short arrow) corresponded to EphB4, the lower bands (arrowheads) a combination of both. Multiple bands may be due to differences in posttranslational modifications, in particular phosphorylation on multiple residues. Altogether, both chimeras appeared to be activated to similar levels by Fc fragments corresponding to cognate ligands. Note a slight activation by ephrinB2 Fc in controls, reflecting the abundance of endogenous receptors for ephrinB2 in the ectoderm.

**Figure S4**

**Eph kinase activity is required for tissue separation.** (A) KD variants of EphA4 (EphA4KD) and EphB4 (EphB4 KD) act as dominant negatives. EphA4KD expression in the mesoderm inhibited tissue separation and failed to rescue EphA4 depletion. Identical results were obtained by expression of EphB4KD in the ectoderm. (B) Ectopic induction of tissue separation between ectoderm explants by ephrinB2 Fc treatment was blocked by expression of KD EphB4. (B’) Induction of separation between mesoderm explants by ephrinB3 Fc treatment was similarly inhibited by expression of EphA4KD. ** indicates *p* < 0.01 (Student’s *t* test) compared to second columns. (C) Inhibition of separation by ephrinB2 depletion in the mesoderm can be rescued by treatment of the ectoderm with soluble ephrinB2 fragments (see Figure 1D). Expression of EphB4KD, however, blocked the ability of ectoderm cells to respond to ephrinB2. Similarly, soluble ephrinB3 Fc could not rescue separation between ephrinB3-depleted ectoderm and EphA4KD-expressing mesoderm. ** indicates *p* < 0.01 (Student’s *t* test) compared to the first columns. “ns,” not significant. (D) Inhibition of Eph phosphorylation. Left panel, EphB4. Control and EphB4KD-expressing ectoderm explants were treated with soluble ephrinB2 Fc fragments. Extracts were prepared and analyzed by immunoblot for p-EphB, total EphB4, and tubulin. Stimulation of EphB phosphorylation by ephinB2 Fc fragments was strongly inhibited by expression of EphB4KD. Right panel, EphA4. Significant phosphorylation of EphA4 was observed in untreated mesoderm explants, consistent with activation by one of its endogenous ligands, ephrinB2 (Figure 2), which is abundantly expressed in the mesoderm (Figure S1). Expression of EphA4KD in the mesoderm strongly decreased the p-EphA signal and failed to rescue p-EphA levels in EphA4MO-coinjected explants. Arrowhead, nonspecific band.

**Figure S5**

**Effect of cadherin levels and ephrin-Eph signaling on separation and tissue cohesion.** (A) Inhibition of separation upon cadherin overexpression and myosin inhibition. Tissue separation was inhibited by cadherin overexpression in the mesoderm but was rescued by increasing Eph signaling by treatment with soluble ephrinB2 Fc fragments. Separation was also strongly inhibited by treatment of wild-type explants with the myosin inhibitor blebbistatin. (B) Cadherin levels are not affected by Eph depletion. Immunofluorescence for C-cadherin of cryosections from whole embryos injected with control or anti-Eph morpholinos. GFP (immunostained in red) was used as the tracer. Note the strong disruption of the ectoderm–mesoderm boundary. (C–E) Tissue cohesion is decreased upon cadherin depletion or ectopic ephrin/Eph expression. Dissociated ectoderm and mesoderm cells were left to reaggregate under mild rotation for 1 h. (C) Effect of cadherin and/or EphB4 depletion on mesoderm reaggregation. (D) Effect of ephrin/Eph ectopic expression on mesoderm or ectoderm reaggregation. Ectoderm-specific ephrinB3 and EphB4 were expressed in the mesoderm, and mesoderm-specific ephrinB2 and EphA4 in the ectoderm. (E) Quantification of reaggregation. Two criteria were used, which gave similar results: the average aggregate area, which reflects the extent of aggregation, and area/perimeter ratio, which integrates both the size of the aggregates and their degree of compaction. Results from individual experiments were normalized using wild-type ectoderm/mesoderm as the reference (1.0) to account for batch-to-batch variation.

**Figure S6**

**Dose response of Eph activation by soluble ephrins.** Ectoderm explants were incubated for 30 min in the presence of different concentrations of ephrin-Fc fragments. Total extracts were analyzed by Western blot for p-Tyrosin levels. Phosphorylated Ephs represent a prominent band around 110 kDa (arrow) (see also Figure 2B). Samples were standardized for protein amount using β-catenin levels (as plasma membrane marker, also compared to total protein on Ponceau Red staining, not shown). (A) Example. (B) Average data from three experiments after subtraction of the endogenous signal, calculated from control condition (Fc). Curve fitting (one phase association) using GraphPad gave similar approximate Kds of ~0.5–5 nM for both ephrins. Note that the curve for ephrinB2 was peculiar. Although its shape was compatible with calculation of the curve, it did not plateau, a feature that was reported in other cases and is not yet explained. The apparent Kd for ephrinB2 should be considered as a “global” affinity for all its ectodermal receptors (mostly EphBs). The apparent Kd for ephrinB3 can be considered to correspond to its Kd for EphA4, as it does not interact with EphBs (Figure 2D).

**Figure S7**

**Simulation of ephrin/Eph signaling in dorsal ectoderm and mesoderm and at the boundary.** (A) Principle of the simulation: the total signal output due to all the interactions between ephrins and Eph receptors at the tissue interface is computed (red double arrow), also taking into account the involvement of these molecules at homotypic contacts with surrounding cells in each tissue (pale double arrows). (A’) Diagrams of all the high affinity interactions between ephrins and Eph receptors at different cell contacts. Relative concentrations are symbolized by the size of the boxes, whereas the thickness of the red lines represents the relative intensities of the individual signals. (B and C) Apparent affinities and concentrations used for the simulation (basal values). (D) Effect of varying the range of concentrations and affinities on output for the stage 10.5 dorsal boundary. Each range of concentration was obtained by multiplying the values of table C by the indicated value. Selected affinities were varied as indicated. All other values were as in table B. The condition marked by a star corresponds to the basal values of tables B and C. (E) Results from Figure 4A, included for comparison. (F–J) Functional effect of manipulating ephrin and Eph levels: comparison of results from the separation assay (taken from Figures 2A, 3, S2A, and S2B) and of the corresponding simulation, using basal parameter values. The simulated boundary outputs are expressed as 100% of intensity signal at control ectoderm–mesoderm contacts.

**Figure S8**

**Effect of ephrin/Eph gain- and loss-of-function on ventral ectoderm–mesoderm separation.** (A) Summary of ephrin/Eph expression in stage 11 ventral tissues. The major differences compared to the dorsal side (Figure 1A) were the mesoderm enrichment of ephrinB1 and the even distribution of EphB4. (B) Inhibition of separation. Separation was assayed as in Figure 1B, but using ventral ectoderm and mesoderm (ventral lip) explants, dissected from stage 11 embryos. Separation was significantly impaired upon depletion of ephrinB3 and EphB2 on the ectoderm side, and for their corresponding partners EphA4 and ephrinB1 on the mesoderm side of the boundary. Note the stronger effect of ephrinB1 depletion compared to the results on the dorsal side (Figure S2A), consistent with its shift from an equal to an asymmetric distribution. ** indicate *p* < 0.01 (Student’s *t* test) compared to corresponding controls (white columns). (C) Induction of separation. Control ventral ectoderm explants normally mix. Significant separation was observed upon explant treatment with soluble Fc fragments corresponding to mesoderm-enriched ephrinB1 and B2, but not ephrinB3. EphrinB1-Fc–induced separation was significantly inhibited by EphB2 depletion, but not EphB4 depletion. The result is consistent with EphB2 acting as the preferred receptor for ephrinB1 (Figure 2B).

**Movie S1**

Live imaging of contacts between single ectoderm and mesoderm cells. A mixture of dissociated wild-type ectoderm cells expressing membrane-targeted GFP (green) and mesoderm cells expressing membrane-targeted Cherry (red) was plated on fibronectin. Newly re-established homotypic contacts between cells of the same tissue remained stable. Contacts between ectoderm and mesoderm cells underwent cycles of attachment (arrowheads) and repulsion/detachment (arrows). This movie shows maximum projection of 3 z planes of 0.2 μm distance.

**Movie S2**

Live imaging of contact between single ectoderm and mesoderm cells treated with blebbistatin. Mesoderm cells (red) and ectoderm cells (green) treated with 100 μM blebbistatin formed stable heterotypic contacts.

**Movie S3**

Live imaging of contacts between single ectoderm and mesoderm cells overexpressing cadherin. Mesoderm cells overexpressing cadherin (red) and ectoderm cells (green) maintained stable heterotypic contacts.

**Movie S4**

Live imaging of contacts between control mesoderm cells. Examples of formation of stable contacts between wild-type mesoderm cells (arrowheads).

**Movie S5**

Live imaging of contacts between cadherin-depleted mesoderm cells. Mesoderm cells injected with cadherin MO failed to establish stable contacts. The cell on the bottom right attaches (arrowheads) and detaches (arrows) several times from the neighboring cells. Arrowheads point to attachments, arrows to detachments.

**Movie S6**

Live imaging of contacts between cadherin/EphB4-depleted mesoderm cells. Mesoderm cells were prepared from embryos coinjected with cadherin MO and EphB4MO. Cells formed contact (arrowheads) and failed to retract. This demonstrates that the repulsion observed between cadherin MO cells was due to ephrin–Eph signaling.

**Movie S7**

Live imaging of contacts between mesoderm cells overexpressing ephrinB3 and EphB4. Ectopic coexpression of “ectodermal” ephrinB3 and EphB4 induced cycles of attachment and detachments between mesoderm cells. Arrowheads mark sites of contact and arrows subsequent retractions between the cell on the right side of the field and the two neighboring cells in the middle.
